# Lactational Enrofloxacin Exposure Promotes Offspring Lipid Metabolic Disorder and Adiposity Involving Gut Microbiota-Derived LPS and PPARγ Signaling

**DOI:** 10.3390/microorganisms14091964

**Published:** 2026-09-05

**Authors:** Yuhui Li, Yating Cao, Zhongyu Zhang, Xia Wang, Anqi Wang, Jigang Zhang, Yehao Liu

**Affiliations:** 1 School of Life and Health Sciences, Environmental Engineering, Hefei University, Hefei 230601, China; liyuhui@hfuu.edu.cn (Y.L.);; 2 School of Public Health, Anhui Medical University, Hefei 230032, China

**Keywords:** enrofloxacin, early-life, gut microbiota, adipose, lipopolysaccharide

## Abstract

Early-life antibiotic exposure is a critical environmental trigger for developmental metabolic disorders and long-term obesity risk. Lactational enrofloxacin exposure exerts potential metabolic programming toxicity, but its long-term effects and underlying mechanisms remain poorly defined. This study aimed to investigate the persistent influences of lactational enrofloxacin exposure on postnatal growth, lipid metabolism and adipogenesis in mouse offspring and explore the potential gut microbiota-associated regulatory mechanism. A combined in vivo and in vitro approach was utilized. In vivo mouse models with lactational enrofloxacin exposure were established to assess growth phenotypes, serum lipid profiles, adipose morphology, and gut microbial composition via high-throughput sequencing. Antibiotic cocktail treatment was performed to deplete gut microbiota. In vitro 3T3-L1 cell models and pharmacological inhibition assays were used to investigate the downstream signaling pathway. Lactational enrofloxacin exposure was associated with disrupted postnatal growth, persistent weight gain, fat accumulation and progressive dyslipidemia in offspring. Adipose expansion was mainly attributed to adipocyte hyperplasia. It triggered sustained gut dysbiosis, increased Gram-negative *Proteobacteria*, and elevated circulating lipopolysaccharide (LPS) levels (systemic endotoxemia). Microbiota depletion largely reversed these metabolic abnormalities. In vitro, LPS facilitated preadipocyte differentiation and lipid deposition, consistent with activation of a PPARγ-dependent mechanism (putatively via TLR4). Our findings suggest that lactational enrofloxacin exposure is associated with persistent fat accumulation and lipid metabolism disorders in offspring and support a key role for the gut microbiota–LPS–PPARγ signaling pathway, with TLR4 as the putative upstream receptor, in this process. These findings provide insights into the developmental metabolic toxicity of early-life fluoroquinolone exposure and offer a theoretical basis for preventing early-onset metabolic diseases.

## 1. Introduction

Obesity and dyslipidemia have evolved into prevalent chronic metabolic disorders worldwide, imposing heavy burdens on public health systems [1,2,3]. Mounting epidemiological and preclinical evidence supports the developmental programming hypothesis, which states that adverse environmental stimuli during early life can permanently reshape physiological metabolism and elevate the lifelong risk of metabolic diseases in offspring, even if the initial exposure ceases after the neonatal period [4]. Among diverse early-life environmental hazards, antibiotic overexposure has drawn growing research attention: broad-spectrum antibiotics disrupt the stability of intestinal microecology in immature organisms, thereby triggering long-term metabolic dysregulation in adulthood [5,6].

Enrofloxacin, a synthetic fluoroquinolone antibiotic, is extensively utilized in livestock breeding, aquaculture, and veterinary clinical treatment due to its broad antibacterial spectrum and low cost [7,8]. Residual enrofloxacin is frequently detected in animal-derived food, drinking water, and maternal breast milk, resulting in unavoidable low-dose lactational exposure for neonatal mammals [9,10]. Previous toxicological studies have confirmed that perinatal antibiotic exposure disturbs intestinal microbial homeostasis and promotes fat accumulation in offspring [11,12]. Nevertheless, existing research lacks systematic exploration of the persistent metabolic programming effects induced by lactational enrofloxacin exposure, including stage-specific alterations in postnatal growth, adipose cellular remodeling, and progressive dyslipidemia across weaning and adulthood. More critically, the complete gut microbiota-dependent molecular cascade linking maternal enrofloxacin administration during lactation to offspring obesity and lipid metabolic dysfunction remains insufficiently characterized.

The gut microbiota serves as a vital signaling bridge connecting early antibiotic intervention and host metabolic homeostasis [13,14]. Gram-negative bacteria within the intestinal flora secrete lipopolysaccharide (LPS), a membrane endotoxin capable of triggering systemic low-grade inflammation and disrupting lipid metabolism upon translocation into the circulation [15,16]. Abnormally elevated circulating LPS (metabolic endotoxemia) is widely recognized as a core intermediate driver of adipocyte hyperplasia, hypercholesterolemia, and obesity [17,18]. Multiple studies have demonstrated that LPS activates the TLR4/PPARγ signaling axis, the master regulatory pathway governing preadipocyte differentiation and lipogenic gene transcription [19,20,21]. However, it remains unclear whether lactational enrofloxacin exposure reshapes gut microbiota composition to expand Gram-negative taxa, boost endogenous LPS production and intestinal translocation, and subsequently drive sustained lipid metabolic damage in offspring via the TLR4/PPARγ pathway. Furthermore, microbiota depletion rescue experiments and in vitro adipocyte functional validation are still required to verify the causal relationship of the gut microbiota-LPS-TLR4/PPARγ axis in mediating enrofloxacin-induced developmental metabolic toxicity.

To fill the above research gaps, the present study constructed a lactational enrofloxacin exposure mouse model combined with an antibiotic cocktail microbiota-depletion intervention group to systematically characterize in vivo phenotypic changes in offspring, including postnatal growth trajectories, body fat deposition, serum lipid profiles, adipocyte morphological remodeling, gut microbial community shifts, and endogenous LPS concentrations at weaning and adulthood. We further performed in vitro functional assays using 3T3-L1 preadipocytes to confirm the direct pro-adipogenic effect of LPS and validate the regulatory role of the TLR4/PPARγ signaling pathway via pharmacological inhibition. Collectively, this work aims to: (1) clarify the long-term developmental metabolic hazards of lactational enrofloxacin exposure on offspring adiposity and lipid homeostasis; (2) identify gut microbiota as the indispensable upstream mediator of enrofloxacin-triggered metabolic programming; (3) uncover LPS as the key microbial-derived intermediate molecule linking intestinal dysbiosis to persistent dyslipidemia and obesity susceptibility; and (4) elucidate the downstream TLR4/PPARγ molecular mechanism driving LPS-induced adipocyte differentiation and lipid accumulation. This research establishes a full toxicological signaling cascade of early fluoroquinolone exposure and provides novel experimental evidence for preventing childhood and adult metabolic diseases originating from neonatal antibiotic exposure.

## 2. Materials and Methods

### 2.1. Animals and Experimental Design

Specific pathogen-free (SPF) C57BL/6J female mice (8–10 weeks old, weight 20–22 g) and male mice (8–10 weeks old, weight 22–25 g) were purchased from Beijing Vital River Laboratory Animal Technology Co., Ltd. (Beijing, China). All mice were housed in a controlled environment with a 12 h light/dark cycle (light on at 8:00 AM, light off at 8:00 p.m.), constant temperature (22 ± 2 °C) and relative humidity (50 ± 5%), and were provided with a standard chow diet and sterile drinking water ad libitum. All animal experiments were approved by the Animal Ethic Committee of Anhui Medical University (protocol code LLSC20220647, 15 May 2022) and were conducted in accordance with the Guide for the Care and Use of Laboratory Animals.

Female mice were mated with male mice at a ratio of 2:1. After confirming pregnancy (by visual observation of vaginal plugs and weight gain), pregnant mice were randomly divided into two groups: the control group (n = 8) and lactational antibiotic exposure group (n = 8). From the 1st day of lactation to the 21st day of lactation (weaning), the antibiotic exposure group named the Enro group was given drinking water containing enrofloxacin (0.27 mg/mL. With an average body weight of 30 g and daily water intake of approximately 4 mL, the corresponding daily enrofloxacin exposure was approximately 36 mg/kg body weight in mice. Using the standard human equivalent dose (HED) conversion based on body surface area (Km_mouse = 3, Km_human = 37; conversion factor = 0.081), this dose corresponds to a HED of approximately 2.9 mg/kg/day (equivalent to ~174 mg/day for a 60 kg adult). This dosage was designed to model a high-exposure scenario of lactational enrofloxacin intake for mechanistic investigation of developmental metabolic toxicity and is well below the acute oral toxicity threshold in mice (LD50 > 5000 mg/kg), while the control group was given normal sterile drinking water. It should be noted that this dose (36 mg/kg/day) represents the maternal drinking water exposure dose, and the actual enrofloxacin dose received by the offspring via breast milk was not directly quantified in this study, as breast milk enrofloxacin concentration and pup milk intake were not measured. The offspring exposure dose via lactational transfer may differ from the maternal dose and warrants further investigation. To explore the contribution of gut microbiota to lipid metabolic dysfunction in offspring, an antibiotic (AB)-treated group was established. Specifically, offspring in this group were born to enrofloxacin-exposed dams and thus received identical lactational enrofloxacin exposure as the Enro group; from weaning (postnatal day 21) onward, they were administered an antibiotic cocktail supplemented in sterile drinking water (1 g/L ampicillin, 0.5 g/L vancomycin, and 1 g/L metronidazole) continuously until the end of the experiment (16 weeks of age). The antibiotic-containing drinking water was refreshed every 2–3 days to ensure drug stability. Therefore, the AB-treated group represents a microbiota-depletion rescue condition applied to enrofloxacin-exposed offspring (i.e., an Enro+AB design), not an independent antibiotic-only group. The three groups compared in this study are: control (no enrofloxacin, no AB), Enro (lactational enrofloxacin exposure only), and AB (lactational enrofloxacin exposure + post-weaning antibiotic cocktail). This treatment was applied to deplete the intestinal commensal microbiota and to verify whether the metabolic phenotypes induced by lactational enrofloxacin exposure are gut microbiota-dependent.

After weaning (3 weeks old), offspring mice were separated from their mothers and housed in groups (4–5 mice per cage, same sex), and all offspring were fed with a standard chow diet and normal drinking water until 16 weeks old (adulthood). Offspring mice were randomly selected from each maternal group for subsequent detection at two time points: weaning stage (3 weeks old) and adulthood (16 weeks old). To minimize potential litter effects, no more than two pups per litter were used for any single detection assay, and offspring were randomly selected across all 8 dams per group with equal male/female representation. Litter was treated as a random factor in key statistical analyses to account for potential non-independence of pups from the same dam. Each detection endpoint included offspring from all 8 independent litters per group whenever possible. After weaning, offspring were housed 4–5 mice per cage with same-sex littermates or non-littermates as randomly assigned; mice from different litters were co-housed whenever possible to minimize cage effects. Both litter and cage (nested within litter) were considered as random factors in the linear mixed-effects models for key analyses. Male and female offspring were pooled with approximately equal 1:1 representation per group; sex was included as a covariate in the mixed-effects models to statistically account for potential sex differences. Formal sex-stratified analysis was not performed due to sample size constraints, and this is acknowledged as a study limitation.

The number of offspring used for each detection index is specified as follows: longitudinal body weight tracking (growth curve), n = 10 per group; body fat percentage, n = 6–8 per group; serum lipid profiles (TC, TG, LDL-C, HDL-C), n = 6 per group; adipose tissue histomorphometry, n = 6 per group; gut microbiota 16S rRNA sequencing, n = 5 per group; fecal LPS quantification, n = 4 per group; and serum LPS quantification, n = 6 per group. For all indices requiring euthanasia, offspring were randomly selected from each maternal group, with approximately equal male/female representation to avoid gender bias. For in vitro experiments (Oil Red O staining, intracellular TG quantification, qPCR), each experiment was independently repeated at least three times with n = 3–4 replicates per group.

### 2.2. Detection of Body Fat Rate and Blood Lipid Indicators

One-week-old pups were individually marked using picric acid. Body weights of all offspring were recorded weekly throughout the rearing period. Partial animals from each group were sacrificed at weaning (3 weeks of age) and adulthood (16 weeks of age), respectively. Inguinal adipose tissue and gonadal adipose tissue were dissected and weighed immediately after dissection. The body fat percentage was calculated according to published references using the following formula: body fat percentage = (weight of inguinal fat pad + weight of gonadal fat pad)/body weight of the mouse according to the reference [22].

At 16 weeks old (adulthood), mice were fasted for 12 h, and blood samples were collected from the orbital venous plexus into sterile centrifuge tubes. Blood samples were centrifuged at 3000 rpm for 15 min at 4 °C to separate serum, which was stored at −80 °C until detection. Serum lipid indicators, including total cholesterol (TC), triglyceride (TG), low-density lipoprotein cholesterol (LDL-C) and high-density lipoprotein cholesterol (HDL-C), were detected using commercial enzyme-linked immunosorbent assay (ELISA) kits (Nanjing Jiancheng Bioengineering Institute, Nanjing, China) according to the manufacturer’s protocols. The absorbance was measured at 450 nm using a microplate reader (Bio-Rad, Hercules, CA, USA), and the concentration of each indicator was calculated based on the standard curve.

### 2.3. Observation of Adipocyte Size and Count

Both weaning mice and 16-week-old mice were included in the adipocyte morphological observation experiment. After anesthesia, the mice were sacrificed by cervical dislocation, and epididymal white adipose tissue (eWAT) and inguinal white adipose tissue (iWAT) were rapidly isolated and weighed. The harvested adipose tissue samples were fixed in 4% paraformaldehyde solution at 4 °C for 24 h, followed by gradient ethanol dehydration (70%, 80%, 90%, 95%, and 100%), paraffin embedding, and continuous sectioning at a thickness of 5 μm. The paraffin sections were dewaxed with xylene, rehydrated through a gradient ethanol series, and stained with hematoxylin–eosin (HE) following standard histological staining protocols.

All stained sections from weaning mice and 16-week-old mice were observed under an optical microscope (Olympus BX53, Tokyo, Japan). Five random and non-overlapping fields of view were selected for each section at a magnification of 200×. Microscopic images were captured using a digital camera coupled to the microscope system. Adipocyte size (diameter) and cell number were quantitatively analyzed using Image-Pro Plus v6.0 software (Media Cybernetics, Rockville, MD, USA). Specifically, the average adipocyte diameter was determined by randomly selecting and measuring 50 adipocytes per field, and the estimated total adipocyte number per fat pad was calculated by multiplying the mean adipocyte density (cells/mm^2^, quantified from 5 random fields) by the total cross-sectional area of the corresponding fat pad (mm^2^), as measured from the entire histological section. All morphological analyses were performed uniformly and blindly to ensure experimental consistency and objectivity.

### 2.4. Gut Microbiota Analysis by High-Throughput Sequencing

Fresh fecal samples were collected from offspring mice at 21 days old and 16 weeks old, immediately frozen in liquid nitrogen, and stored at −80 °C until DNA extraction. Total genomic DNA was extracted from fecal samples (100 mg per sample) using a QIAamp PowerFecal Pro DNA Kit (Qiagen, Hilden, Germany) according to the manufacturer’s instructions. The concentration and purity of extracted DNA were detected using a NanoDrop 2000 spectrophotometer (Thermo Fisher Scientific, Waltham, MA, USA), and DNA integrity was verified by 1% agarose gel electrophoresis.

The V3-V4 hypervariable region of the bacterial 16S rRNA gene was amplified by polymerase chain reaction (PCR) using the specific primers 338F and 806R. PCR amplification was performed in a 25 μL reaction system containing 12.5 μL of 2× Taq Plus Master Mix (Vazyme, Nanjing, China), 1 μL of each primer (10 μM), 2 μL of DNA template (10 ng/μL), and 8.5 μL of sterile double-distilled water. The PCR program was as follows: pre-denaturation at 95 °C for 5 min; 30 cycles of denaturation at 95 °C for 30 s, annealing at 55 °C for 30 s, and extension at 72 °C for 45 s; and final extension at 72 °C for 10 min. PCR products were verified by 1.5% agarose gel electrophoresis, and target bands were purified using a DNA Gel Extraction Kit (Axygen, Union City, CA, USA).

Purified PCR products were quantified using a Qubit 3.0 Fluorometer (Thermo Fisher Scientific, Waltham, MA, USA), and equimolar amounts of each sample were pooled to construct a sequencing library. The library was sequenced on an Illumina MiSeq PE300 platform (Illumina, San Diego, CA, USA) by Shanghai Personalbio Technology Co., Ltd. (Shanghai, China). Raw sequencing data were filtered to remove low-quality reads, chimeras and adapter sequences using Trimmomatic v0.39 and UCHIME v11.0.667 software. Operational taxonomic units (OTUs) were clustered at 97% sequence similarity using UPARSE v11.0.667 software. The commissioned analysis included taxonomic composition profiling at the phylum and family levels, as well as alpha-diversity index calculation. Beta-diversity statistics and formal differential-abundance testing were not included in the analysis scope.

The efficacy of antibiotic cocktail-mediated gut microbiota depletion was verified by quantitative PCR (qPCR) targeting the universal bacterial 16S rRNA gene; detailed methods and results are provided in the Supplementary Material.

### 2.5. Detection of LPS Levels

Serum and fecal LPS levels were detected at 21 days old and 16 weeks old using a mouse LPS ELISA kit (Shanghai Enzyme-linked Biotechnology Co., Ltd., Shanghai, China) according to the manufacturer’s instructions. Briefly, serum samples were diluted 1:10 (fecal samples were diluted 1:1000) with sample dilution buffer, and 100 μL of diluted samples, standard solutions (0, 10, 20, 40, 80, 160 pg/mL) and blank control were added to the corresponding wells of the ELISA plate, respectively. The plate was incubated at 37 °C for 90 min, then washed 5 times with washing buffer. Next, 100 μL of horseradish peroxidase (HRP)-labeled secondary antibody was added to each well, and the plate was incubated at 37 °C for 60 min. After washing 5 times again, 100 μL of substrate solution (TMB) was added to each well, and the plate was incubated at 37 °C in the dark for 15 min. Finally, 50 μL of stop solution was added to terminate the reaction, and the absorbance was measured at 450 nm using a microplate reader. The concentration of LPS in the samples was calculated based on the standard curve.

### 2.6. Cell Culture and Treatment

3T3-L1 preadipocytes were purchased from the Wuhan Servicebio Technology Co., Ltd. (Wuhan, China). The cells were cultured in Dulbecco’s Modified Eagle Medium (DMEM, Gibco, Grand Island, NY, USA) supplemented with 10% fetal bovine serum (FBS, Gibco, Grand Island, NY, USA) and 1% penicillin–streptomycin (Gibco, USA) at 37 °C in a humidified atmosphere containing 5% CO_2_. The medium was changed every 3 days, and cells were passaged when they reached 80–90% confluence using 0.25% trypsin-EDTA (Gibco, USA). For all adipogenic differentiation experiments, 3T3-L1 preadipocytes were seeded in 6-well plates at a density of 2 × 10^5^ cells per well, grown to 100% confluence, and then incubated for an additional 2 days (day 0 of differentiation) before induction. Adipogenic differentiation was induced using a standard MDI differentiation cocktail consisting of complete DMEM supplemented with 0.5 mM 3-isobutyl-1-methylxanthine (IBMX, Sigma-Aldrich, St. Louis, MO, USA), 1 μM dexamethasone (Sigma-Aldrich, USA), and 10 μg/mL insulin (Sigma-Aldrich, USA). After 48 h (day 2), the induction medium was replaced with maintenance medium (complete DMEM containing 10 μg/mL insulin), which was refreshed every 2 days until day 8. LPS (Sigma-Aldrich, USA) was dissolved in sterile phosphate-buffered saline (PBS) and diluted to the desired concentrations with culture medium.

For gene expression analysis, LPS was added to the differentiation medium at final concentrations of 4 μg/mL (low dose) or 20 μg/mL (high dose) at the onset of differentiation (day 0), and cells were collected after 48 h of treatment (day 2 of differentiation) for qPCR detection. This experiment was designed to assess the effect of LPS on the early molecular events of adipogenic differentiation. For terminal differentiation assays, LPS (4 μg/mL or 20 μg/mL) was present in both the induction medium (day 0–2) and the maintenance medium (day 2–8) throughout the entire differentiation period, with the medium (containing fresh LPS) being replaced every 2 days. At day 8 of differentiation, cells were harvested for Oil Red O staining and intracellular triglyceride quantification. Control cells were differentiated in parallel without LPS supplementation in all experiments.

At day 8 of differentiation, cells were washed twice with PBS and fixed with 4% paraformaldehyde for 30 min at room temperature. Fixed cells were then stained with Oil Red O working solution (60% saturated Oil Red O in isopropanol, diluted 3:2 with distilled water and filtered) for 30 min at room temperature. After washing three times with distilled water, stained lipid droplets were imaged under an inverted microscope. For quantitative analysis, the stained intracellular lipids were eluted with 100% isopropanol for 10 min with gentle shaking, and the absorbance of the eluate was measured at 510 nm using a microplate reader (Bio-Rad, Hercules, CA, USA). For intracellular triglyceride (TG) quantification, cells were washed twice with PBS and lysed with RIPA lysis buffer (Beyotime, Shanghai, China) at day 8 of differentiation. Intracellular TG levels were quantified using a commercial triglyceride ELISA kit (Nanjing Jiancheng Bioengineering Institute, Nanjing, China) according to the manufacturer’s instructions. The absorbance was measured at 450 nm using a microplate reader, and TG concentrations were calculated based on a standard curve. The TG concentration was normalized to the total protein content of each sample, determined using a BCA protein assay kit (Beyotime, Shanghai, China).

### 2.7. qPCR Detection of Target Genes

Total RNA was extracted from 3T3-L1 cells using Trizol reagent (Invitrogen, Carlsbad, CA, USA) according to the manufacturer’s instructions. The concentration and purity of RNA were detected using a NanoDrop 2000 spectrophotometer, and RNA integrity was verified by 1% agarose gel electrophoresis. Reverse transcription was performed to synthesize complementary DNA (cDNA) using a PrimeScript RT Reagent Kit with gDNA Eraser (Takara, Beijing, China), following the manufacturer’s protocol. The reverse transcription program was: 42 °C for 15 min, 85 °C for 5 s, and 4 °C for storage.

qPCR was performed using a SYBR Premix Ex Taq II Kit (Takara) on a LightCycler^®^ 96 Instrument (Roche, Basel, Switzerland). The reaction system (20 μL) consisted of 10 μL of SYBR Premix Ex Taq II, 0.4 μL of each forward and reverse primer (10 μM), 2 μL of cDNA template, and 7.2 μL of sterile double-distilled water. The qPCR program was: pre-denaturation at 95 °C for 30 s; 40 cycles of denaturation at 95 °C for 5 s; annealing at 60 °C for 30 s; and a melting curve analysis from 60 °C to 95 °C to verify the specificity of PCR products.

The target genes were selected based on their roles in adipocyte proliferation and differentiation: peroxisome proliferator-activated receptor γ (PPARγ), CCAAT/enhancer-binding protein α (C/EBPα), proliferating cell nuclear antigen (PCNA), cyclin D1 (CCND1) and tumor necrosis factor α (TNF-α). Glyceraldehyde-3-phosphate dehydrogenase (GAPDH) was used as the internal reference gene. The primer sequences are listed in Table S1. The relative expression level of each target gene was calculated using the 2^−ΔΔCt^ method, and the expression level in the control group was set as 1.

### 2.8. Statistical Analysis

All data are presented as mean ± standard deviation (mean ± SD). For two-group comparisons, independent-samples *t*-tests were used. For comparisons among three groups, one-way ANOVA coupled with Tukey’s post hoc test was used to correct for multiple comparisons. Longitudinal body weight data were analyzed using repeated-measures two-way ANOVA (group × time), followed by Sidak’s post hoc test for individual time point comparisons. To account for potential non-independence of pups from the same dam, litter was included as a random factor in linear mixed-effects models for key analyses, and no more than two pups per litter were used for any single assay. A value of *p* < 0.05 was defined as statistically significant. All statistical analyses were conducted using GraphPad Prism 9.0 and R software.

For key in vivo analyses, linear mixed-effects models were implemented using the lme4 package in R (version 4.2.1), with experimental group and sex as fixed factors and litter and cage (nested within litter) as random factors. This approach accounts for the non-independence of pups from the same litter and the potential clustering effects of shared housing. *p*-values for fixed effects were obtained via Satterthwaite’s degrees-of-freedom approximation using the lmerTest package v3.1.

## 3. Results

### 3.1. Lactational Enrofloxacin Exposure Increases Body Fat Accumulation in Offspring Mice

**Figure 1:**
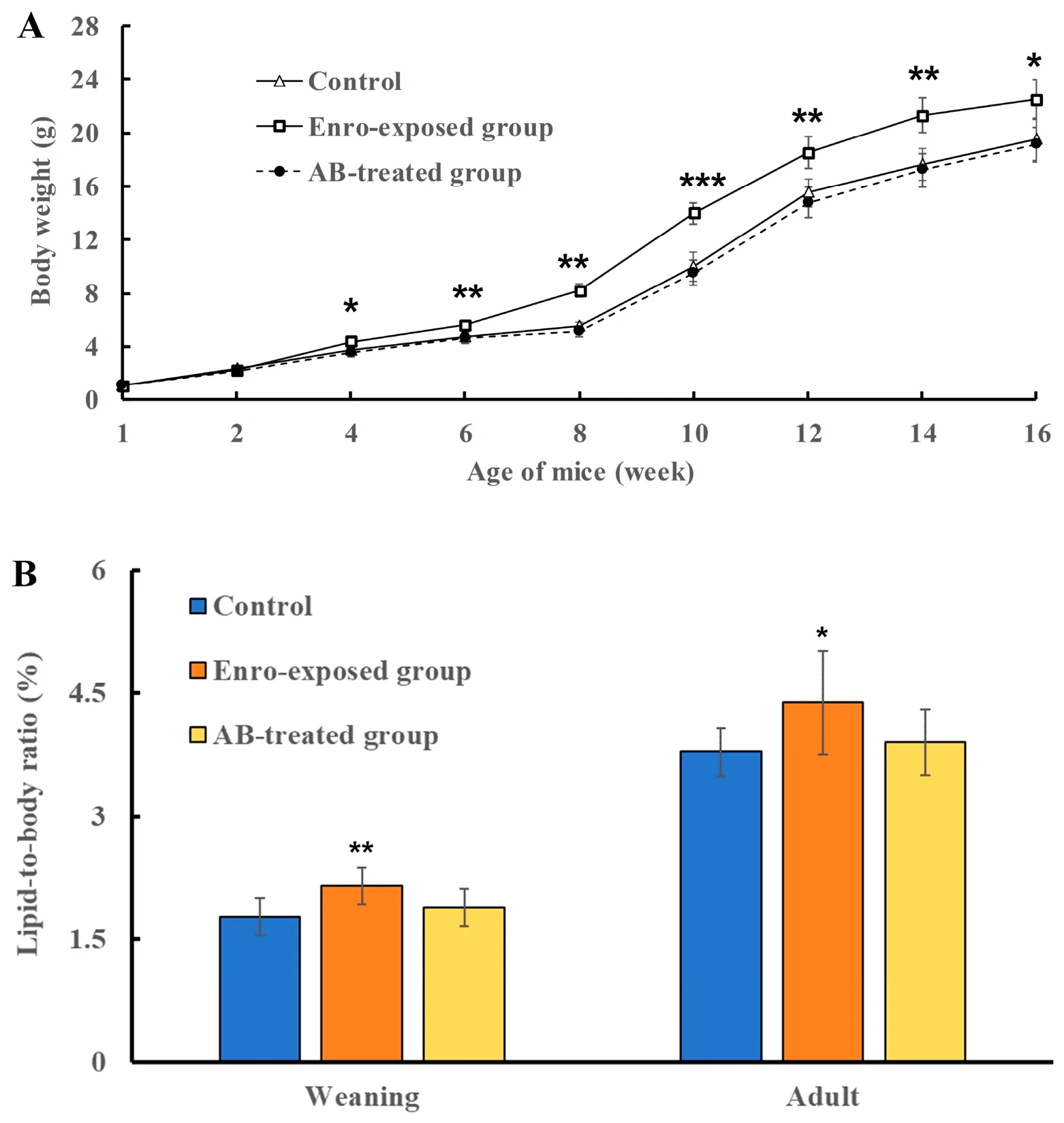
Effects of lactational enrofloxacin exposure on body weight (**A**) and percentage body fat (**B**) in offspring mice. (x ± s, growth curve n = 10; body fat n = 6–8), * *p* < 0.05, ** *p* < 0.01, *** *p* < 0.001.

To assess the effects of lactational enrofloxacin exposure on postnatal growth and adipose deposition in offspring, body weight was recorded biweekly throughout the postnatal period. Adipose tissues (inguinal and gonadal fat) were dissected and weighed at weaning (3 weeks of age) and adulthood (16 weeks of age) to quantify the relative body fat percentage. Growth curve analysis demonstrated that offspring in the enrofloxacin-exposed group attained their maximum growth rate at postnatal week 3, while the control group peaked at postnatal week 4 (Figure 1A). Consistently, enrofloxacin-treated offspring exhibited significantly higher body weight than control mice from postnatal week 3 to week 16. In terms of adiposity, lactational enrofloxacin exposure significantly increased body fat percentage by 20.8% at weaning and 15.9% at adulthood compared with the control group (Figure 1B).

To further determine whether gut microbiota mediates the altered growth and fat deposition phenotypes, an antibiotic cocktail (AB) was used to deplete endogenous gut microbiota in offspring. It should be noted that the AB-treated group consisted of enrofloxacin-exposed offspring that received an antibiotic cocktail from weaning onward (i.e., a rescue design), as detailed in Section 2.1. Notably, depletion of gut microbiota abolished the above phenotypic differences, with no significant differences in body weight and body fat percentage observed between the control and AB-treated groups. Collectively, these results indicate that lactational enrofloxacin exposure disturbs physiological postnatal growth trajectories, increases body weight and fat accumulation in early life, and confers heightened susceptibility to adult obesity in offspring in a gut microbiota-dependent manner.

### 3.2. Lactational Enrofloxacin Exposure Induces Persistent and Progressive Dyslipidemia in Offspring Mice

**Figure 2:**
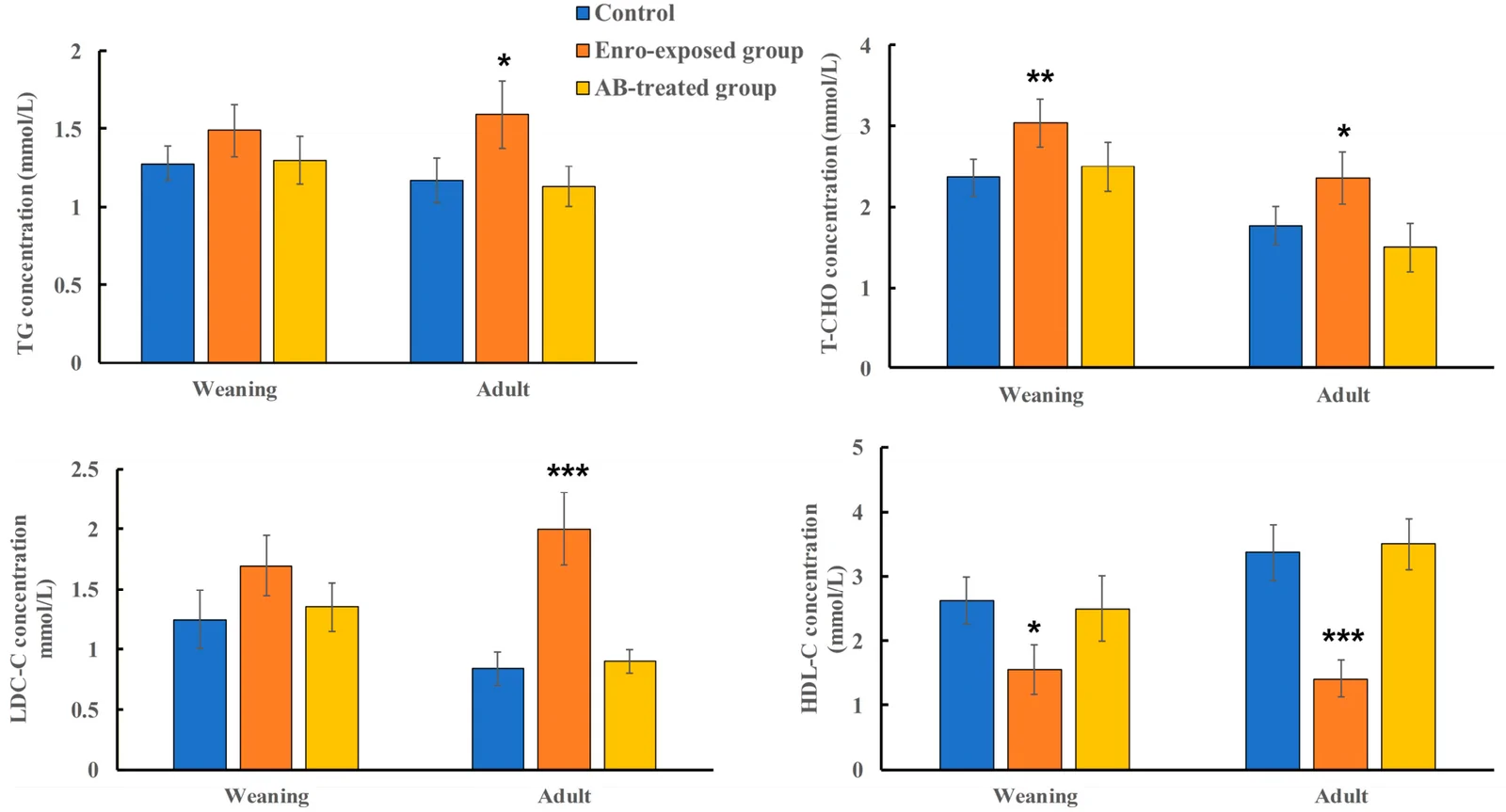
Effects of lactational enrofloxacin exposure on serum T-CHO, TG, LDL-C and HDL-C levels in mouse offspring (mean ± SD, n = 6). * *p* < 0.05, ** *p* < 0.01, *** *p* < 0.001.

Dyslipidemia is a defining metabolic feature tightly linked to the development of obesity. To clarify whether lactational enrofloxacin exposure disrupts lipid homeostasis in progeny, we quantified serum concentrations of total cholesterol (T-CHO), triglycerides (TGs), low-density lipoprotein cholesterol (LDL-C), and high-density lipoprotein cholesterol (HDL-C) in offspring at weaning and adulthood (Figure 2). Biochemical assays revealed that circulating T-CHO levels were significantly elevated by 28.5% at weaning and 33.4% in adulthood in enrofloxacin-exposed pups relative to control counterparts. Serum TG concentrations were comparable across groups at weaning, whereas adult offspring with lactational enrofloxacin exposure exhibited a 35.5% increase in serum TG. Serum LDL-C followed an identical changing pattern to TG: no intergroup divergence was detected at weaning, yet adult enrofloxacin-treated mice presented a striking 138.7% upregulation of LDL-C. Conversely, serum HDL-C concentrations were substantially suppressed in the enrofloxacin group at both developmental time points. Notably, all circulating lipid parameters were indistinguishable between control and antibiotic cocktail (AB)-treated microbiota-depleted groups, suggesting gut microbiota mediates enrofloxacin-triggered lipid metabolic dysfunction. Collectively, these data verify that maternal enrofloxacin administration during lactation elicits persistent and progressive dyslipidemia in offspring: at weaning, the phenotype is characterized by elevated T-CHO and reduced HDL-C and by adulthood it progresses to include additional elevations in TG and LDL-C. This evidence underscores that early-life enrofloxacin intervention is associated with long-lasting disruption to systemic lipid metabolism in progeny.

### 3.3. Lactational Enrofloxacin Exposure Promotes Adipocyte Hyperplasia in Offspring Adipose Tissue

**Figure 3:**
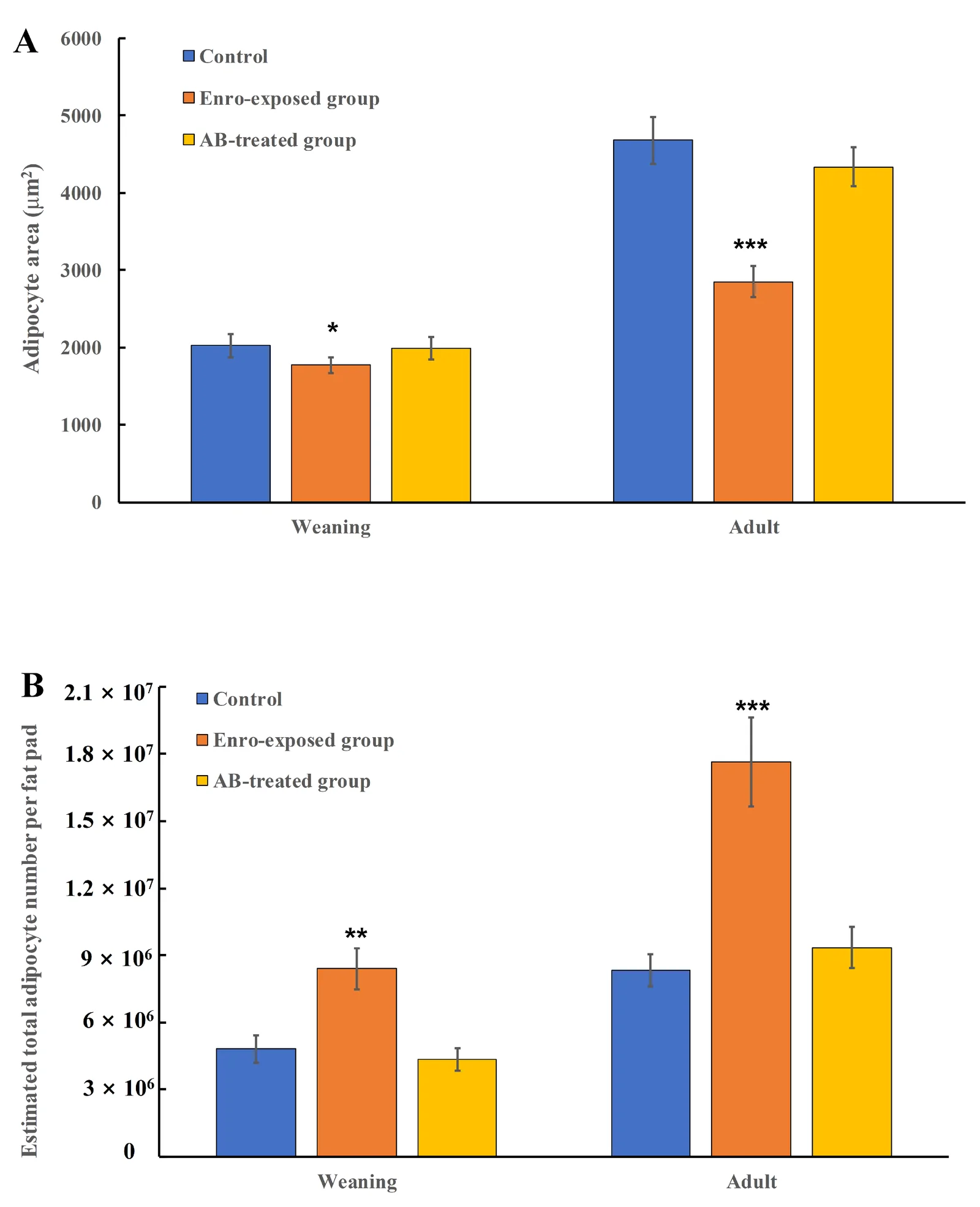
Effects of lactational enrofloxacin exposure on adipocyte area (**A**) and number (**B**) in mouse offspring (mean ± SD, n = 6). The reduced mean adipocyte diameter together with the increased cell count indicates adipocyte hyperplasia. * *p* < 0.05, ** *p* < 0.01, *** *p* < 0.001.

Adipose tissue expansion linked to obesity arises from two principal cellular mechanisms: adipocyte hyperplasia (increased adipocyte quantity) and adipocyte hypertrophy (enlarged single-cell volume). To dissect the cellular basis underlying enrofloxacin-elicited fat accumulation, we conducted morphological quantification to evaluate adipocyte size and abundance within the adipose depots of offspring (Figure 3). Morphometric measurements revealed that mean adipocyte diameter was markedly decreased in enrofloxacin-exposed progeny by 6.3% at weaning and 21.9% at adulthood. In contrast, lactational enrofloxacin exposure triggered a 74.3% rise in adipocyte count at weaning, accompanied by a sustained 112.0% elevation into adulthood. This combination of reduced mean cell diameter and markedly increased cell number indicates that enrofloxacin-induced adipose expansion is driven predominantly by adipocyte hyperplasia (accumulation of numerous small, newly differentiated adipocytes) rather than hypertrophy. Notably, the antibiotic cocktail (AB)-treated microbiota-depleted group exhibited a complete reversal of the adipocyte phenotypic shifts observed in the enrofloxacin group. Representative H&E-stained sections of epididymal white adipose tissue confirmed the quantitative findings, showing smaller adipocytes with increased cell density in the Enro-exposed group compared with controls at both weaning and adulthood, while the AB-treated group exhibited adipocyte morphology comparable to controls (Supplementary Figure S1). Collectively, these data indicate that lactational enrofloxacin exposure is associated with adipose tissue expansion driven primarily by enhanced adipocyte hyperplasia rather than hypertrophy, and that gut microbiota is a necessary mediator of this process (as demonstrated by full reversal in the antibiotic cocktail-treated group). The observed adipocyte hyperplasia may contribute to increased lipid storage capacity and suggests elevated long-term obesity risk in exposed offspring; however, direct assessment of lasting cellular predisposition and lifelong metabolic outcomes requires further investigation beyond the 16-week time point examined in this study.

### 3.4. Lactational Enrofloxacin Exposure Remodels Gut Microbiota Structure in Offspring Mice

**Figure 4:**
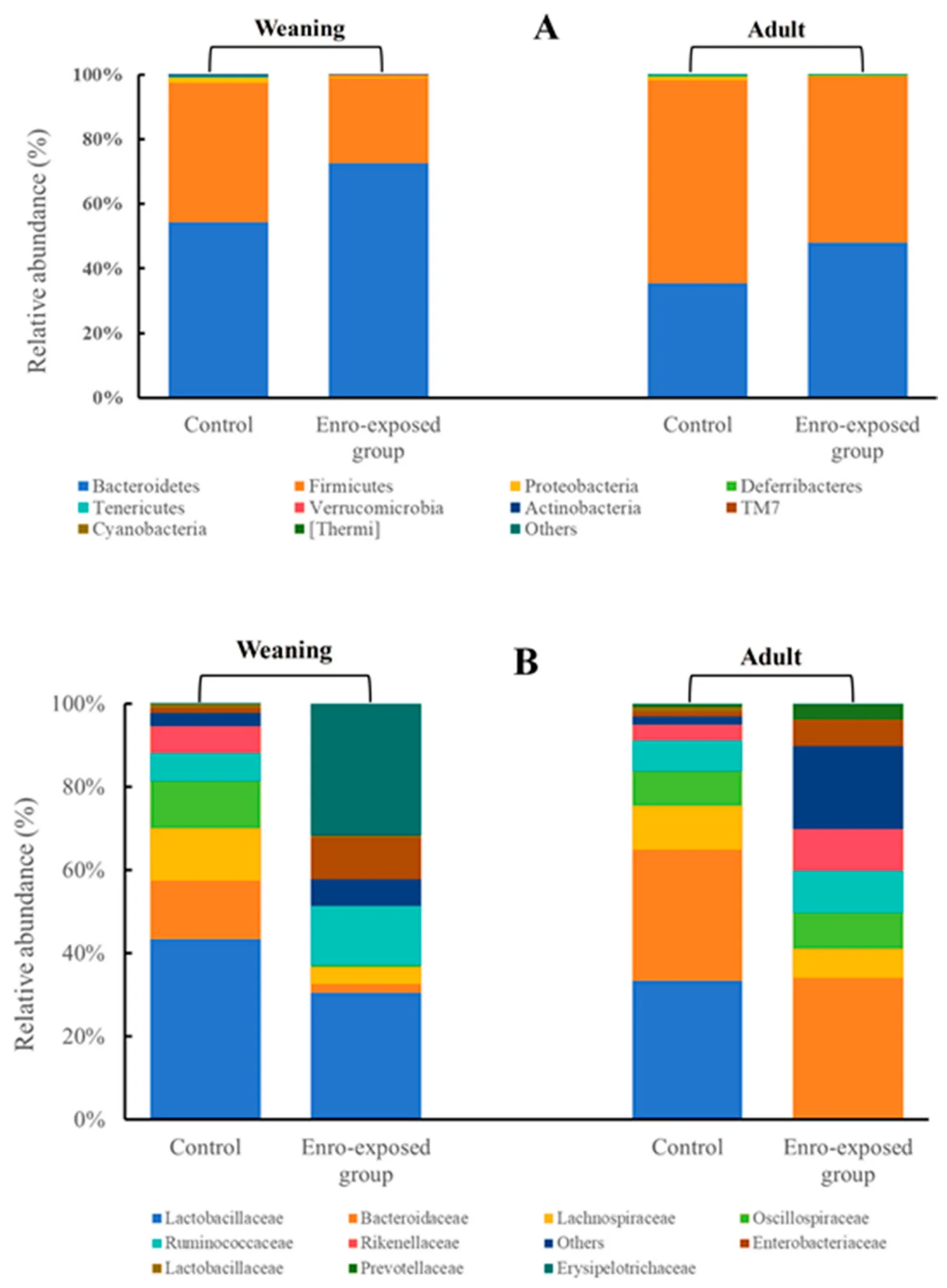
Taxonomic composition based on relative abundance at the phylum (**A**) and family (**B**) levels. n = 5 per group per time point.

Obesity is tightly linked to intestinal dysbiosis, and early-life exposure to low-dose antibiotics can reshape the compositional landscape of the gut microbiota. To characterize disparities in gut microbial community architecture between enrofloxacin-exposed offspring and untreated controls, we conducted high-throughput profiling to assess intestinal microbial diversity in weanling and adult offspring. Alpha diversity analysis revealed that the Shannon index was significantly lower in the Enro-exposed group than in controls at both weaning (2.6 vs. 3.8, *p* < 0.05) and adulthood (3.9 vs. 5.6, *p* < 0.01). Notably, the AB-treated group exhibited extremely low Shannon diversity at both time points (0.8 at weaning and 0.5 at adulthood) (Supplementary Figure S2), consistent with effective gut microbiota depletion confirmed by qPCR (Supplementary Figure S3). As presented in Figure 4A, the relative abundance of phylum *Bacteroidetes* surged to 72.7% in enrofloxacin-treated pups at weaning, substantially exceeding the 54.3% detected in control littermates. Conversely, the phylum *Firmicutes* only accounted for 26.1% of the gut community in the enrofloxacin group, compared with 43.1% in controls. While this intergroup taxonomic divergence was partially alleviated by adulthood, *Bacteroidetes* levels remained significantly enriched in mice with lactational enrofloxacin exposure.

At the family taxonomic rank (Figure 4B), lactational enrofloxacin exposure drastically suppressed the abundance of anti-obesity microbial taxa, namely *Lactobacillaceae*, *Lachnospiraceae,* and *Oscillospiraceae*. Meanwhile, the proportions of pro-obesity families *Erysipelotrichaceae*, *Enterobacteriaceae* and *Ruminococcaceae* were markedly expanded in weanling progeny. Notably, these microbial compositional shifts persisted and were further exacerbated as the offspring matured into adulthood. In addition, the relative load of Gram-negative phylum *Proteobacteria* was significantly elevated in adult mice subjected to lactational enrofloxacin treatment. To further verify the efficacy of gut microbiota depletion in the AB-treated group, total bacterial load was quantified by real-time qPCR targeting the universal bacterial 16S rRNA gene. As shown in Supplementary Figure S3, antibiotic cocktail treatment reduced total fecal DNA concentration by over 95% within one week (from 8.8 to 0.4 ng/mg stool at week 1; *p* < 0.0001) (Figure S3A), and the mean Ct value of the 16S rRNA gene increased from 21.6 at baseline to 38.2 at week 1 (Figure S3B), corresponding to an approximately 100,000-fold reduction in bacterial abundance. This substantial depletion was sustained across all five time points examined from week 1 to week 16, confirming effective and persistent gut microbiota decontamination in the AB-treated group throughout the experimental period. Collectively, these observations reveal that maternal enrofloxacin exposure during lactation induces marked disruption of gut microbial homeostasis in developing offspring, characterized by compositional shifts consistent with an obesity-associated microbial profile (including reduced anti-obesity taxa and expanded obesity-associated and Gram-negative taxa). Whether these microbial changes directly contribute to the metabolic phenotype requires further functional validation, such as fecal microbiota transplantation experiments.

### 3.5. Lactational Enrofloxacin Exposure Elevates Endogenous LPS Levels in Offspring Mice

**Figure 5:**
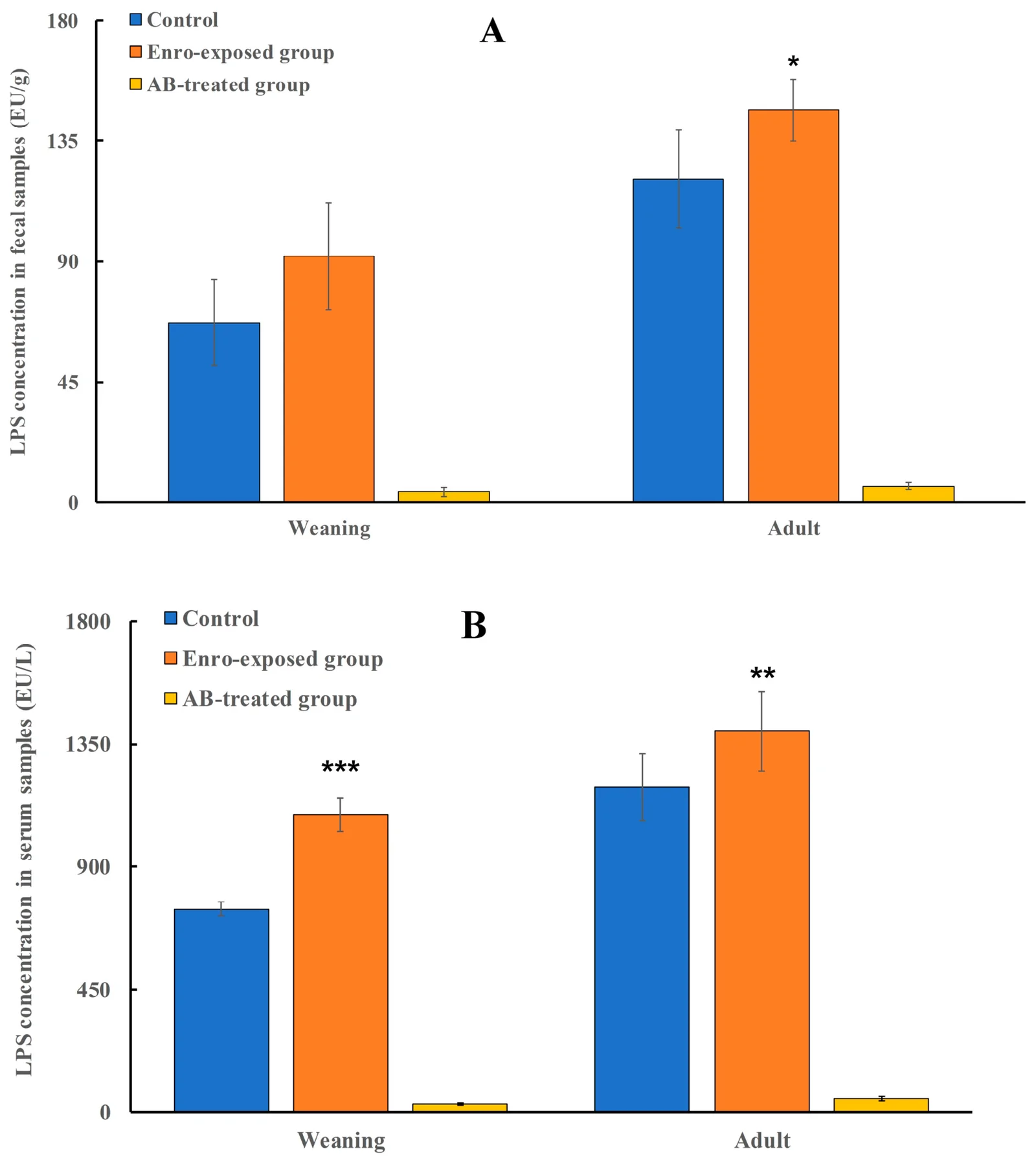
Concentrations of lipopolysaccharide (LPS) in feces (**A**) and serum (**B**) of mouse offspring. Data are presented as mean ± SD. n = 4 for panel A, n = 6 for panel B. * *p* < 0.05, ** *p* < 0.01, *** *p* < 0.001.

LPS, an essential outer membrane constituent of Gram-negative bacteria, acts as a key initiator of metabolic inflammation and dysregulated lipid metabolism. Considering the marked expansion of Gram-negative taxa observed in enrofloxacin-exposed progeny, we further quantified fecal and circulating LPS concentrations using ELISAs. Fecal LPS concentrations remained significantly higher in enrofloxacin-treated pups relative to control littermates from weaning through adulthood (Figure 5A). Consistently, serum LPS abundance was persistently and markedly elevated in the enrofloxacin group at both developmental time points (Figure 5B). In contrast, mice receiving the antibiotic cocktail (AB) to ablate gut microbiota exhibited drastically diminished LPS levels. Collectively, these data indicate that lactational enrofloxacin exposure is associated with gut microbial dysbiosis and increased abundance of Gram-negative bacteria, accompanied by elevated fecal and serum LPS concentrations. These findings are consistent with increased LPS production and enhanced systemic LPS exposure, but the specific mechanisms underlying LPS biosynthesis and intestinal translocation were not directly assessed in this study and warrant further investigation.

### 3.6. LPS Exposure Increases Cellular Lipid Accumulation

**Figure 6:**
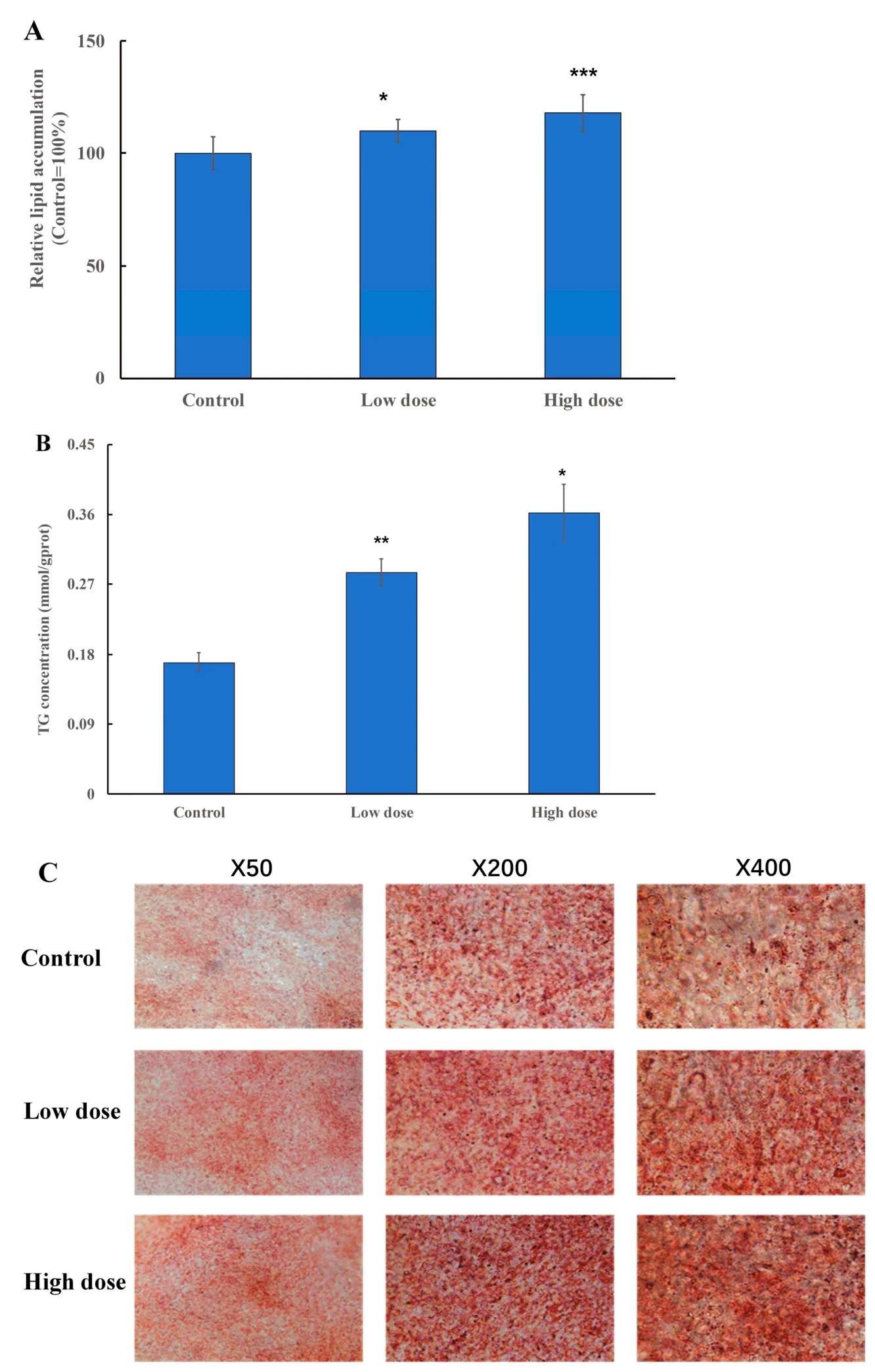
Lipid accumulation in cells treated with gradient concentrations of lipopolysaccharide (LPS). (**A**) Semi-quantitative analysis of Oil Red O staining intensity. (**B**) Quantification of intracellular triglyceride levels. (**C**) Representative images of Oil Red O staining. Data are presented as mean ± SD, n = 3 for panels (**A**,**B**). * *p* < 0.05, ** *p* < 0.01, *** *p* < 0.001.

To explore the regulatory effect of LPS on intracellular lipid accumulation, we performed in vitro experiments using the 3T3-L1 preadipocyte cell line. Morphological observation under an inverted microscope showed that adipogenic differentiation triggered gradual rounding and enlargement of 3T3-L1 cells, accompanied by a progressive increase in the quantity and size of intracellular refractive lipid droplets. Oil Red O staining further verified specific lipid deposition: undifferentiated cells and non-lipid cellular components remained unstained, whereas intracellular lipid droplets in differentiated adipocytes were intensely stained bright red. As presented in Figure 6A, relative to the control group, cells treated with low-dose (4 μg/mL) LPS exhibited a marked increase in lipid droplet abundance, and this lipid-accumulating phenotype was further strengthened following exposure to high-dose (20 μg/mL) LPS.

For quantitative lipid assessment, stained intracellular lipids were eluted with isopropanol, and the absorbance value was spectrophotometrically detected. Consistent with the morphological results (Figure 6B), both the low- and high-dose LPS-treated groups displayed significantly higher absorbance than the control group (*p* < 0.01 and *p* < 0.05, respectively), indicative of elevated intracellular lipid content. To further validate the pro-adipogenic effect of LPS, intracellular triglyceride (TG) levels were quantified via commercial ELISA kits. As shown in Figure 6C, LPS treatment during adipogenic differentiation significantly increased intracellular TG accumulation at both low and high doses (*p* < 0.01 and *p* < 0.05, respectively).

Collectively, these morphological and biochemical results indicate that LPS exposure facilitates 3T3-L1 preadipocyte differentiation and augments intracellular lipid and triglyceride deposition, with a stronger effect observed at the higher LPS concentration (20 μg/mL) compared with the lower concentration (4 μg/mL).

### 3.7. LPS Regulates Adipogenic and Lipogenic Gene Expression Involving PPARγ Signaling

**Figure 7:**
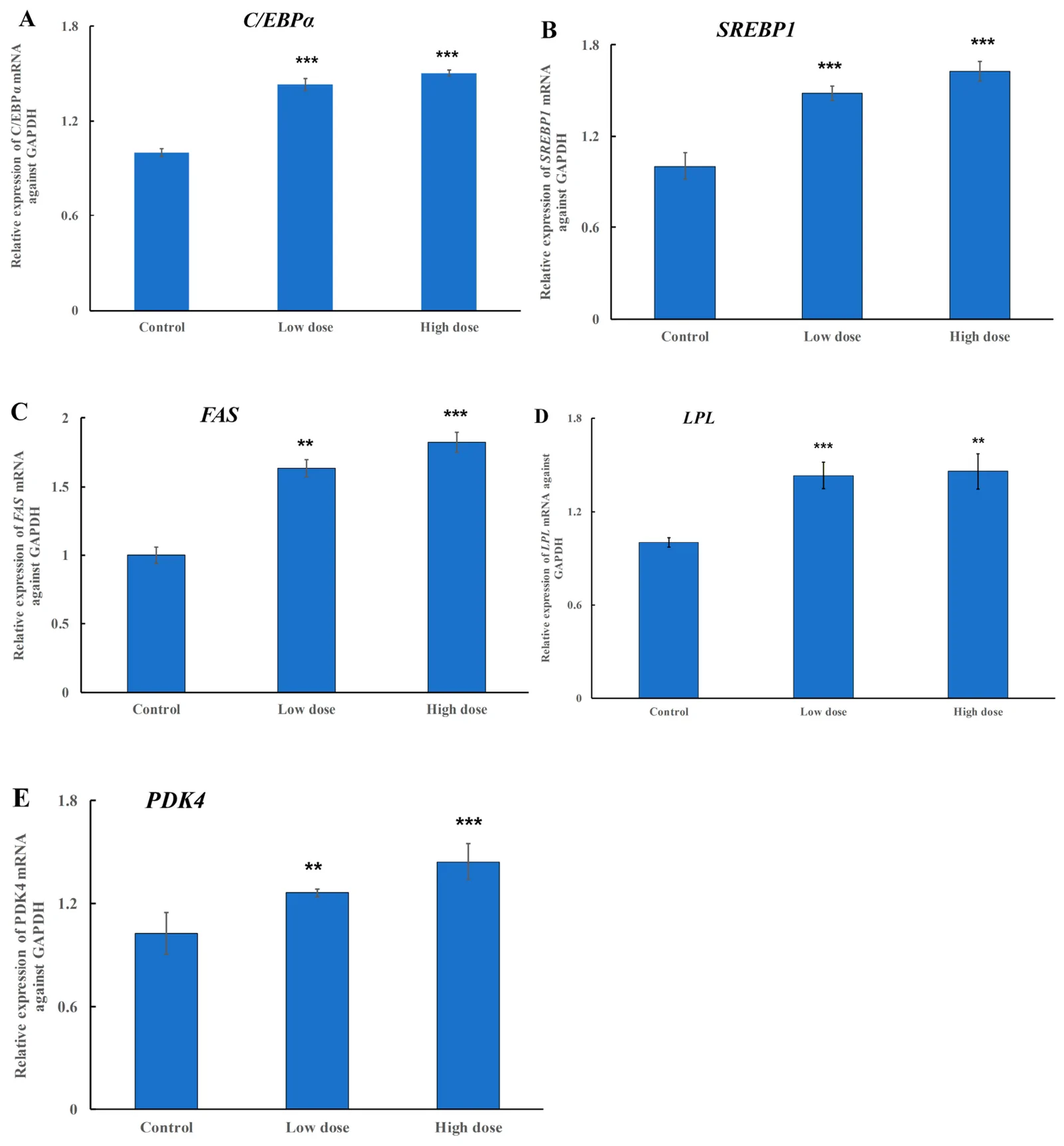
Relative mRNA expression of genes related to cell proliferation, adipogenic differentiation and lipid metabolism under treatment with gradient lipopolysaccharide (LPS) concentrations. (**A**) C/EBPα; (**B**) SREBP1; (**C**) FAS; (**D**) LPL; (**E**) PDK4. All data are expressed as mean ± SD, n = 4. ** *p* < 0.01, *** *p* < 0.001.

To further explore the molecular pathway by which LPS regulates preadipocyte differentiation and lipid metabolism, we quantified the mRNA expression of key genes associated with adipogenic proliferation and differentiation (*PPARγ*, *C/EBPα*, and *SREBP1*), as well as lipid metabolic function, including fatty acid synthase (*FAS*), lipoprotein lipase (*LPL*), and pyruvate dehydrogenase kinase 4 (*PDK4*), via qPCR analysis (Figure 7A–E). Transcriptional profiling demonstrated that both 4 μg/mL and 20 μg/mL LPS treatments significantly upregulated the mRNA levels of *PPARγ*, *C/EBPα*, and *SREBP1*, the master regulators of adipogenesis. Consistent with the pro-adipogenic phenotype, the expression of lipogenic genes *FAS* and *LPL*, as well as the metabolic regulator *PDK4*, was also markedly elevated following LPS stimulation.

**Figure 8:**
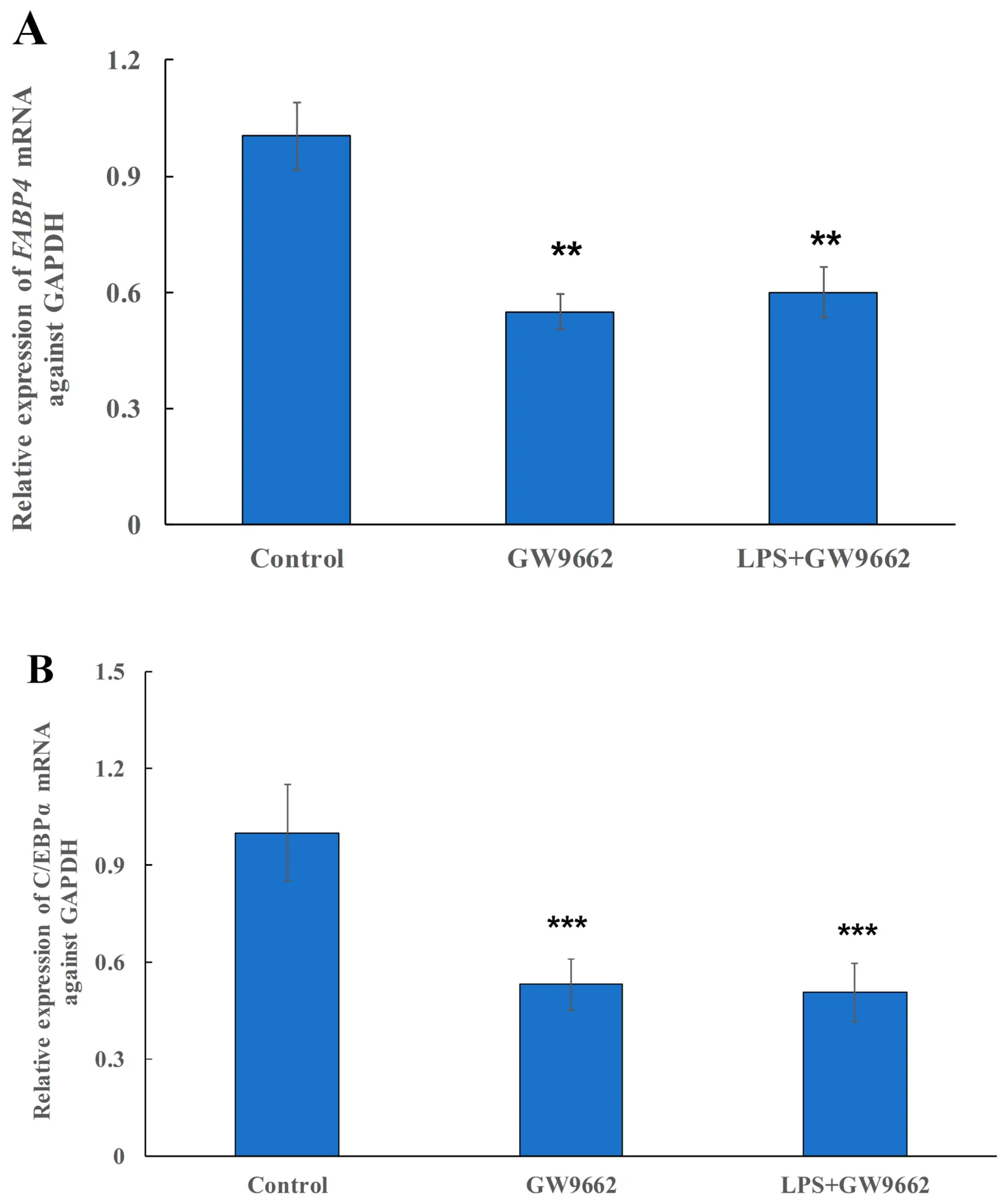
mRNA expression of adipogenic markers FABP4 (**A**) and C/EBPα (**B**) following single or combined treatment with LPS and PPARγ antagonist GW9662. All data are expressed as mean ± SD, n = 4. ** *p* < 0.01, *** *p* < 0.001.

To verify whether PPARγ is required for LPS-regulated adipogenic programming, rescue experiments using the selective PPARγ antagonist GW9662 were performed, and mRNA levels of PPARγ downstream adipogenic markers FABP4 and C/EBPα were quantified. As illustrated in Figure 8, GW9662 treatment alone markedly suppressed FABP4 transcript abundance by 45.2% relative to the vehicle control. Notably, co-stimulation with LPS and GW9662 yielded a 40.0% reduction in FABP4 expression. Consistently, C/EBPα mRNA levels were decreased by 50.3% in both GW9662 monotherapy and LPS plus GW9662 co-treatment groups. Together, these transcriptional data demonstrate that pharmacological inhibition of PPARγ by GW9662 significantly reduces the expression of PPARγ downstream adipogenic targets (FABP4 and C/EBPα), confirming that PPARγ activity is required for the expression of these genes. We note that this specific GW9662 experiment did not include an LPS-only group, and therefore the direct effect of GW9662 on the LPS-induced response cannot be formally calculated from this assay alone. However, when considered together with the observation that LPS upregulates PPARγ mRNA expression (Figure 7A), these findings are consistent with a PPARγ-dependent mechanism underlying LPS-induced adipogenic programming. Given that LPS is the canonical ligand for TLR4, these findings are also consistent with TLR4 acting as the putative upstream receptor; however, direct TLR4 functional validation (e.g., via TLR4-specific inhibition or knockdown) and assessment of PPARγ protein activity were not performed in this study and warrant further investigation.

## 4. Discussion

Early-life antibiotic exposure has been widely recognized as a critical environmental risk factor for developmental metabolic disorders and adult chronic metabolic diseases. Enrofloxacin, a commonly used broad-spectrum fluoroquinolone antibiotic, is frequently detected in environmental media and animal-derived foods, leading to unavoidable lactational exposure in neonatal individuals [9]. However, the long-term metabolic programming effects and underlying molecular mechanisms of lactational enrofloxacin exposure on offspring adiposity and lipid metabolism remain poorly clarified. The present study systematically explored the phenotypic changes, structural microbial alterations, and molecular signaling mechanisms of enrofloxacin-induced adiposity and dyslipidemia based on in vivo animal models and in vitro adipocyte experiments. Our findings suggest that lactational enrofloxacin exposure triggers persistent metabolic dysfunction in offspring via the gut microbiota-LPS-TLR4/PPARγ signaling cascade.

Consistent with the core hypothesis of developmental programming [23], our in vivo results demonstrated that lactational enrofloxacin exposure disrupts the normal postnatal growth trajectory of offspring mice. Offspring in the enrofloxacin-exposed group exhibited advanced peak growth rate, elevated body weight from weaning to adulthood, and significantly increased body fat percentage. These phenotypic changes indicate that early-life enrofloxacin intervention is associated with abnormal early growth acceleration and persistent fat accumulation, suggesting elevated long-term obesity risk in exposed offspring. Notably, further morphological analysis of adipose tissue clarified the cellular mechanism of fat deposition: enrofloxacin exposure reduced mean adipocyte diameter (converted from cell area) but significantly increased adipocyte number throughout development, indicating that adipose tissue expansion induced by lactational enrofloxacin exposure is predominantly driven by adipocyte hyperplasia rather than hypertrophy. This finding differs from the conventional obesity model dominated by adipocyte hypertrophy [24,25], revealing a unique cellular programming pattern of antibiotic-induced developmental obesity. The observed adipocyte hyperplasia may contribute to increased lipid storage capacity; however, whether this represents an enduring cellular predisposition requires further investigation beyond the 16-week time point examined.

Dyslipidemia is a key intermediate metabolic phenotype linking early adverse exposure to adult obesity [26,27]. The present study verified that lactational enrofloxacin exposure induces persistent and progressive lipid metabolism disorders in offspring. Specifically, enrofloxacin exposure was associated with sustained elevation of serum total cholesterol throughout development, while triglyceride and low-density lipoprotein cholesterol levels were only significantly increased in adulthood, accompanied by a persistent reduction in high-density lipoprotein cholesterol. The stage-specific changes in blood lipid profiles suggest that early enrofloxacin exposure initiates latent lipid homeostasis imbalance in weaning offspring, which gradually deteriorates and develops into typical persistent and progressive dyslipidemia in adulthood. This progressive metabolic damage is consistent with the increased adiposity observed in exposed offspring and highlights the long-term delayed toxic effect of lactational enrofloxacin exposure on developmental metabolism.

Gut microbiota dysbiosis is a core regulatory hub connecting antibiotic exposure and host metabolic dysfunction [13,28]. Consistent with previous studies confirming that low-dose antibiotics alter intestinal microecology [29], our microbial profiling results demonstrated that lactational enrofloxacin exposure induces profound and persistent gut microbiota remodeling in offspring. At the phylum level, enrofloxacin exposure persistently increased the relative abundance of Bacteroidetes and decreased Firmicutes in both weanling and adult offspring, altering the classic Firmicutes/Bacteroidetes ratio closely associated with obesity. At the family level, enrofloxacin significantly depleted obesity-protective microbial taxa (*Lactobacillaceae*, *Lachnospiraceae*, and *Oscillospiraceae*) while enriching obesity-associated *Erysipelotrichaceae*, *Enterobacteriaceae* and *Ruminococcaceae*, and these microbial disorders were aggravated with aging [30,31,32,33,34]. Moreover, the abundance of Gram-negative Proteobacteria was significantly upregulated in adult exposed mice [35]. These microbial structural changes indicate that lactational enrofloxacin exposure reshapes the gut microecology toward a profile consistent with an obesity-associated microbial signature, which may represent an important upstream factor contributing to subsequent metabolic disorders. Whether these microbial changes directly exert obesogenic effects requires further functional validation, such as fecal microbiota transplantation experiments.

Lipopolysaccharide (LPS), the outer membrane component of Gram-negative bacteria, is a key pro-inflammatory and metabolic disruptive mediator derived from gut microbiota [36]. Combined with the increased abundance of Gram-negative bacteria, our further detection of endogenous LPS levels revealed a critical mechanistic link between microbial dysbiosis and metabolic dysfunction. Fecal LPS levels showed a significant increase both at the weaning and adult stages in enrofloxacin-exposed mice, which was closely related to the increased total abundance of intestinal Gram-negative bacteria. More importantly, circulating serum LPS levels were persistently and significantly increased in exposed offspring throughout development. These findings are consistent with increased LPS production and enhanced systemic LPS exposure; however, the specific mechanisms underlying LPS biosynthesis and intestinal translocation were not directly assessed in this study. This sustained metabolic endotoxemia may act as a crucial intermediate signal bridging lactational antibiotic exposure and long-term lipid metabolism disorders. To further verify the role of gut microbiota in enrofloxacin-mediated metabolic programming, an antibiotic cocktail intervention assay was performed to deplete endogenous intestinal flora in offspring mice, which is a mature and widely recognized approach for validating gut microbiota–host metabolic interactions in preclinical studies. Consistent with extensive reports in the literature [37,38], broad-spectrum antibiotic cocktail treatment effectively eliminated the majority of intestinal aerobic and anaerobic microorganisms in offspring mice, resulting in a nearly complete depletion of total intestinal microbiota and a remarkable reduction in the abundance of Gram-negative Proteobacteria and other core microbial taxa. Notably, gut microbiota depletion largely abolished the enrofloxacin-induced elevations in fecal and serum LPS levels in offspring mice, confirming that the sustained endotoxemia observed in enrofloxacin-exposed offspring is dependent on gut microbial dysbiosis. Furthermore, antibiotic-mediated microbiota elimination significantly reversed the abnormal adipocyte hyperplasia, excessive fat accumulation, and persistent and progressive dyslipidemia phenotypes triggered by lactational enrofloxacin exposure. These collective microbiota depletion results validate that gut microbiota is a necessary mediator for the maintenance and progression of enrofloxacin-induced developmental metabolic disorders, and the LPS derived from dysregulated intestinal flora is the key functional mediator linking early enrofloxacin exposure to subsequent lipid metabolic dysfunction and elevated obesity risk.

In vitro 3T3-L1 preadipocyte experiments further verified the direct regulatory effect of LPS on adipocyte differentiation and lipid accumulation, supporting the involvement of the gut microbiota–LPS axis in enrofloxacin-induced adiposity. Oil Red O staining and quantitative detection of intracellular triglycerides demonstrated that LPS intervention significantly increases intracellular lipid droplet formation and triglyceride deposition and promotes the differentiation of preadipocytes, with a stronger effect observed at the higher LPS concentration (20 μg/mL) compared with the lower concentration (4 μg/mL). At the molecular level, LPS exposure significantly upregulated the mRNA expression of adipogenic transcription factors (PPARγ, C/EBPα, SREBP1) and lipogenic functional genes (FAS, LPL, PDK4). Pharmacological verification with the PPARγ inhibitor GW9662 further confirmed that LPS regulates adipocyte differentiation and lipid metabolism via a PPARγ-dependent mechanism [39]. Given that LPS is the canonical ligand for TLR4, these findings are consistent with TLR4 acting as the upstream receptor mediating LPS-induced PPARγ activation; however, direct TLR4 functional validation (e.g., via TLR4-specific inhibition or knockdown) was not performed in this study and warrants further investigation. Collectively, these in vitro results complement the in vivo phenotypic findings and clarify the downstream molecular mechanism by which LPS may mediate developmental fat accumulation and lipid dysregulation.

In summary, the present study supports a toxicological mechanism chain in which lactational enrofloxacin exposure is associated with persistent gut microbiota dysbiosis in offspring, increased Gram-negative bacteria abundance and elevated circulating LPS levels, activation of PPARγ-dependent adipogenic signaling (putatively via TLR4), enhanced adipocyte hyperplasia and lipogenic gene expression, and ultimately early abnormal fat accumulation, persistent and progressive dyslipidemia and increased adult obesity risk. Antibiotic-mediated microbiota depletion demonstrates that gut microbiota is necessary for these metabolic phenotypes, and in vitro experiments are consistent with LPS promoting adipogenic differentiation via a PPARγ-dependent mechanism; however, these approaches support but do not fully prove the sufficiency of the entire in vivo causal cascade. Nevertheless, this study still has certain limitations. First, the dynamic changes in gut microbiota and LPS levels during the whole developmental stage were only detected at weaning and adulthood, and continuous time point monitoring can be supplemented in subsequent studies. Second, this study focused on the phenotypic and molecular changes in adipose tissue, and the systemic metabolic inflammatory response caused by LPS endotoxemia needs further exploration. In addition, targeted microbial intervention experiments are required to further verify the causal relationship between key microbial taxa and metabolic disorders. Fourth, although our family-level microbiota analysis indicated suppression of *Lachnospiraceae* and *Lactobacillaceae* (major SCFA-producing taxa), the actual concentrations of fecal and circulating short-chain fatty acids (butyrate and acetate) were not directly quantified in this study; targeted GC-MS metabolomic profiling will be included in future investigations to elucidate the interplay between anti-inflammatory SCFA metabolites and pro-inflammatory LPS in developmental metabolic programming. Fifth, the present study focused exclusively on metabolic phenotypes and did not assess potential effects on brain function or neurobehavior via the gut–brain axis, despite the observed microbial dysbiosis and metabolic endotoxemia having clear relevance to neuroinflammatory pathways; comprehensive neurobehavioral and neuroinflammatory assessments will be a priority in future studies. Sixth, the formal sufficiency of the dysbiotic microbiota was not tested by fecal microbiota transplantation (FMT), which represents a limitation of this study. Future FMT experiments will be needed to definitively establish the sufficiency of the enrofloxacin-remodeled microbiota and to dissect LPS-dependent versus independent microbial contributions. Seventh, the actual enrofloxacin exposure dose received by the offspring via breast milk was not directly quantified in this study. The calculated 36 mg/kg/day represents the maternal drinking water exposure dose, and the offspring dose via lactational transfer may differ. Future studies should directly measure enrofloxacin concentrations in breast milk and quantify actual offspring exposure to more precisely characterize the dose–response relationship. Eighth, formal sex-stratified analysis was not performed owing to sample size constraints. Future studies with larger sample sizes are needed to explore potential sex-dependent effects.

## 5. Conclusions

This study systematically investigates the developmental metabolic toxicity of lactational enrofloxacin exposure on offspring mice and its potential underlying molecular mechanism. Lactational enrofloxacin exposure is associated with disrupted postnatal growth homeostasis, persistent fat accumulation and progressive dyslipidemia in offspring, and elevated long-term obesity risk. Mechanistically, early enrofloxacin intervention is associated with persistent gut microbiota structural remodeling, characterized by altered phylum-level microbial ratio, depletion of beneficial anti-obesity taxa and enrichment of obesity-associated flora, as well as increased abundance of Gram-negative *Proteobacteria*. The dysregulated gut microecology is accompanied by elevated fecal and circulating LPS concentrations, consistent with increased LPS production and systemic endotoxemia; however, the specific mechanisms of LPS biosynthesis and intestinal translocation were not directly assessed. Subsequent in vitro experiments confirm that LPS can directly promote adipogenic differentiation and lipid deposition in 3T3-L1 preadipocytes via a PPARγ-dependent mechanism, as validated by the PPARγ antagonist GW9662. Given that LPS is the canonical ligand for TLR4, these findings are consistent with TLR4 as the putative upstream receptor, although direct TLR4 functional validation was not performed. Antibiotic-mediated microbiota depletion reverses the metabolic abnormalities, demonstrating that gut microbiota are necessary for the observed phenotype. In conclusion, our findings suggest that lactational enrofloxacin exposure is associated with offspring adiposity and lipid metabolism disorders and support a key role for the gut microbiota–LPS–PPARγ signaling pathway (with TLR4 as the putative upstream receptor) in this process. However, these approaches support but do not fully prove the sufficiency of the entire in vivo causal cascade, which warrants further investigation using targeted approaches such as fecal microbiota transplantation or TLR4-deficient models. This study provides novel experimental evidence for understanding the developmental metabolic toxicity of early antibiotic exposure and offers a theoretical basis for preventing childhood and adult metabolic diseases associated with early enrofloxacin exposure.

## Data Availability

The data presented in this study are included in the article. Further inquiries can be directed to the corresponding author.

## Supplementary Materials

The following supporting information can be downloaded at https://www.mdpi.com/article/10.3390/microorganisms14091964/s1, Table S1, Primer sequences. Figure S1, Representative hematoxylin and eosin (H&E) staining of epididymal white adipose tissue (eWAT). Figure S2, Alpha diversity (Shannon index) of gut microbiota. Figure S3, Verification of gut microbiota depletion by antibiotic cocktail treatment.

## References

[1] Donini L.M., Ballesteros-Pomar M.D., Batsis J.A., Boirie Y., Cappellari G.G., Poggiogalle E., Barazzoni R. (2026). Obesity is always a clinically relevant chronic disease. Eat. Weight Disord..

[2] Kim K., Hong M., Lee S.G., Chang H.J., Kim T.H. (2025). Trends in Health Expenditures for Chronic Disease Management: Hypertension, Diabetes Mellitus, and Dyslipidemia from 2013 to 2020. Yonsei Med. J..

[3] Henney A.E., Wilding J.P. (2026). The Global Obesity Epidemic: Epidemiology, Health Burden and Policy Priorities. Eur. Cardiol..

[4] Fioretto M.N., Barata L.A., Ribeiro I.T., Maciel F.A., Mattos R., de Souza P.V., Portela L.M.F., Dos Santos S.A.A., Scarano W.R., Justulin L.A. (2025). Early and long-term effects of maternal protein restriction on offspring organs and systems: Insights from the developmental origins of health and disease (DOHaD). Biogerontology.

[5] Guarner F., Bustos Fernandez L., Cruchet S., Damião A., Maruy Saito A., Riveros Lopez J.P., Rodrigues Silva L., Valdovinos Diaz M.A. (2024). Gut dysbiosis mediates the association between antibiotic exposure and chronic disease. Front. Med..

[6] Shan L., Gao M., Pan X., Li W., Wang J., Li H., Tian H. (2022). Association between fluoroquinolone exposure and children’s growth and development: A multisite biomonitoring-based study in northern China. Environ. Res..

[7] Xi F. (2024). The enrofloxacin pollution control from fish to environment. Mar. Pollut. Bull..

[8] López-Cadenas C., Sierra-Vega M., García-Vieitez J.J., Diez-Liébana M.J., Sahagún-Prieto A., Fernández-Martínez N. (2013). Enrofloxacin: Pharmacokinetics and metabolism in domestic animal species. Curr. Drug Metab..

[9] Gao R., Chen Y., Su Y., Waterhouse G.I.N., Qiao X., Sun Y., Xu Z. (2026). Development of a portable electrochemical sensor based on STAB-GN for simultaneous detection of chloramphenicol and enrofloxacin in foods. Anal. Chim. Acta.

[10] Wang W., Chen S., Fu Y., Hong Y., Tang C., Zou L., Tang J., He L., Liu S., Hu K. (2026). Construction of N/S CQDs@Fe-TCPP Nanocatalyst-Induced Electrochemical Sensors for Rapid and Sensitive Detection of Enrofloxacin Residues in Milk. Foods.

[11] Thomas S.P., Denizer E., Zuffa S., Best B.M., Bode L., Chambers C.D., Dorrestein P.C., Liu G.Y., Momper J.D., Nizet V. (2023). Transfer of antibiotics and their metabolites in human milk: Implications for infant health and microbiota. Pharmacotherapy.

[12] Ainonen S., Paalanne M., Ronkainen E., Pokka T., Honkila M., Kajantie E., Paalanne N., Ruuska-Loewald T. (2026). Early-life antibiotic exposure and the risk of overweight and obesity in children. Pediatr. Res..

[13] Zhu B., Fu C., Fan D., Wang Y., Sheng P., Feng J., Wang X. (2026). The impact of antibiotic exposure on obesity and metabolic phenotypes via the gut microbiota. Front. Microbiol..

[14] van Wesemael A.J., Klomp K., Malinowska A.M., van Kaam A.H., de Meij T.G.J., Belzer C., Niemarkt H.J. (2026). Early empiric antibiotic exposure affects gut microbiota development of very preterm infants. Pediatr. Res..

[15] Tume R., El Sherbiny S., Bono R., Gautier T., Pais de Barros J.P., Meroño T. (2025). The balance between proinflammatory, “bad”, and immunomodulatory, “good”, lipopolysaccharide for understanding gut-derived systemic inflammation. Front. Immunol..

[16] Tang J., Yang Z.H., Li W., Liu H., Lucero D., Sviridov D., White O., Kun J., Mukouyama Y.S., Remaley A.T. (2026). Cytoprotective role of octacosanol in lipopolysaccharide-induced inflammation. Front. Immunol..

[17] Mehboodi M., Namdari M.P., Abdollahi Z., Mobarezi Z., Kiani M., Chamani F., Khanbabaie H., Rabiei S., Maleki M.H., Sanati H. (2025). The relationship between increased levels of microbiota-derived lipopolysaccharide in obesity and the pathophysiology of neurodegenerative diseases. Microb. Pathog..

[18] Hersoug L.G., Møller P., Loft S. (2018). Role of microbiota-derived lipopolysaccharide in adipose tissue inflammation, adipocyte size and pyroptosis during obesity. Nutr. Res. Rev..

[19] Bando K., Endo Y. (2026). Zoledronate enhances lipopolysaccharide-induced inflammation via toll-like receptor 4 upregulation. J. Dent. Sci..

[20] Gai Z., Wang R., Xiao L., Yan S., Min C., Gong G., Zhao J. (2026). Isorhamnetin attenuates lipopolysaccharide-induced myocardial inflammatory injury by inhibiting PPARγ and preserving mitochondrial homeostasis. Phytomedicine.

[21] Shantaram D., Hoyd R., Blaszczak A.M., Antwi L., Jalilvand A., Wright V.P., Liu J., Smith A.J., Bradley D., Lafuse W. (2024). Obesity-associated microbiomes instigate visceral adipose tissue inflammation by recruitment of distinct neutrophils. Nat. Commun..

[22] Kvedaras M., Minderis P., Cesanelli L., Cekanauskaite A., Ratkevicius A. (2021). Effects of fasting on skeletal muscles and body fat of adult and old C57BL/6J mice. Exp. Gerontol..

[23] Vaiserman A., Koliada A., Lushchak O. (2018). Developmental programming of aging trajectory. Ageing Res. Rev..

[24] Rocha S., Proença C., Carvalho F., Fernandes E. (2026). Adipose Tissue Plasticity and Adipogenesis: From Cellular Mechanisms to Therapeutic Targets in Obesity. Curr. Obes. Rep..

[25] Liu K., Chen S., Cao M., Xu X., Jiang L., Liu Z., Lai J., Pan X., Zhang Y., Xu L. (2026). Salecan improves diet-induced obesity and hepatic steatosis by regulating citrate cycle intermediates. Int. J. Biol. Macromol..

[26] Kim E., Jeong S.M., Lee D.H., Rezende L.F.M., Giovannucci E.L. (2026). Comparison of obesity indices for discriminating hypertension, diabetes, dyslipidemia, and metabolic syndrome according to sex and age groups. Obes. Res. Clin. Pract..

[27] Zeljkovic A., Vekic J., Stefanovic A. (2024). Obesity and dyslipidemia in early life: Impact on cardiometabolic risk. Metabolism.

[28] Jiang C.Y., Huang Z.L., Liu J.L., Cen S.R., Lu R.M., Wei C.B., Meng H.Y., Xu Q.J. (2026). Nervonic Acid Prevents HFD-Induced Metabolic Dysfunction and Is Associated with Gut Microbiota Remodeling. Metabolites.

[29] He Z., Deng N., Zheng B., Gu Y., Chen J., Li T., Liu R.H., Yuan L., Li W. (2023). Apple peel polyphenol alleviates antibiotic-induced intestinal dysbiosis by modulating tight junction proteins, the TLR4/NF-κB pathway and intestinal flora. Food Funct..

[30] Salazar-Jaramillo L., de la Cuesta-Zuluaga J., Chica L.A., Cadavid M., Ley R.E., Reyes A., Escobar J.S. (2024). Gut microbiome diversity within Clostridia is negatively associated with human obesity. mSystems.

[31] Zeng H., Larson K.J., Cheng W.H., Bukowski M.R., Safratowich B.D., Liu Z., Hakkak R. (2020). Advanced liver steatosis accompanies an increase in hepatic inflammation, colonic, secondary bile acids and Lactobacillaceae/Lachnospiraceae bacteria in C57BL/6 mice fed a high-fat diet. J. Nutr. Biochem..

[32] Xu K., Guo Y., Wang Y., Ren Y., Low V., Cho S., Ping L., Peng K., Li X., Qiu Y. (2023). Decreased Enterobacteriaceae translocation due to gut microbiota remodeling mediates the alleviation of premature aging by a high-fat diet. Aging Cell.

[33] Muegge B.D., Kuczynski J., Knights D., Clemente J.C., González A., Fontana L., Henrissat B., Knight R., Gordon J.I. (2011). Diet drives convergence in gut microbiome functions across mammalian phylogeny and within humans. Science.

[34] Kaakoush N.O. (2015). Insights into the Role of Erysipelotrichaceae in the Human Host. Front. Cell Infect. Microbiol..

[35] Wang H., Wang Y., Wu H., Shen C., Li Y., Bai B., Sun X., Liu Y., Zhang Q., Shi L. (2026). High-fat diet-induced obesity-related hypertension via altered gut microbiota-mediated histone butyrylation. Sci. China Life Sci..

[36] Zhang X., Tian X., Wang Y., Yan Y., Wang Y., Su M., Lv H., Li K., Hao X., Xing X. (2024). Application of lipopolysaccharide in establishing inflammatory models. Int. J. Biol. Macromol..

[37] Li J., Pu F., Peng C., Wang Y., Zhang Y., Wu S., Wang S., Shen X., Li Y., Cheng R. (2022). Antibiotic cocktail-induced gut microbiota depletion in different stages could cause host cognitive impairment and emotional disorders in adulthood in different manners. Neurobiol. Dis..

[38] Panda S.S., Pattnaik T., Aich P. (2025). Antibiotic Cocktail: An Excellent Tool to Probe Physiology by Perturbing Gut Microbiota: A Mice Model. Curr. Microbiol..

[39] Sharma S., Pathirajage K.S., Johnson T., Battu J.R., Dasgupta S. (2025). GW-9662, a peroxisome proliferator-activated receptor γ (PPARγ) inhibitor, impairs early embryonic development in zebrafish. Comp. Biochem. Physiol. C Toxicol. Pharmacol..

