# Supplementary material for: Lactational Enrofloxacin Exposure Promotes Offspring Lipid Metabolic Disorder and Adiposity Involving Gut Microbiota-Derived LPS and PPARγ Signaling
Source: Microorganisms. 2026 Sep 5;14(9):1964. doi: 10.3390/microorganisms14091964 (PMC13609559; doi:10.3390/microorganisms14091964)
Supplement: Supplementary file 1 [file microorganisms-14-01964-s001.zip › microorganisms-4492529-supplementary.pdf]

Table S1. Primer sequences.

Gena name	Upstream primer sequence	Downstream primer sequence
C/EBPα	CAAGAACAGCAACGAGTACCG	GTCACTGGTCAACTCCAGCAC
FAS	GGAGGTGGTGATAGCCGGTAT	TGGGTAATCCATAGAGCCCAG
LPL	GGGAGTTTGGCTCCAGAGTTT	TGTGTCTTCAGGGGTCCTTAG
PDK4	AAACCCAAGCCACATTGGAAGTA	CGCAGAGCATCTTTGCACAC
SREBP1	GATGTGCGAACTGGACACAG	CATAGGGGGCGTCAAACAG
GAPDH	TTCAACGGCACAGTCAAGG	CTCAGCACCAGCATCACC

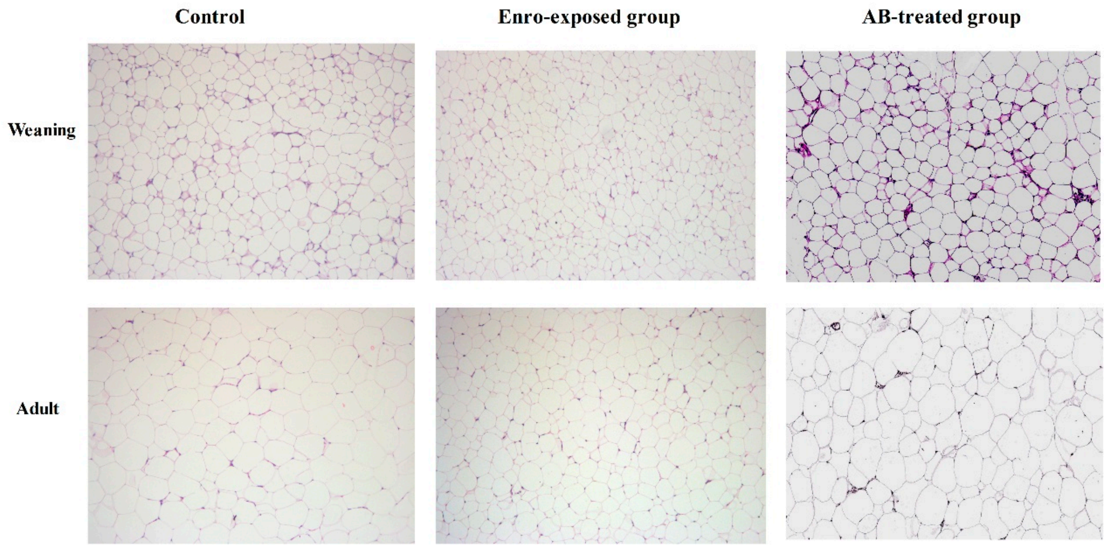

Figure S1. Representative hematoxylin and eosin (H&E) staining of epididymal white adipose tissue (eWAT) from Control, Enro-exposed, and AB-treated offspring at weaning (3 weeks) and adulthood (16 weeks). n = 6 per group at each time point.

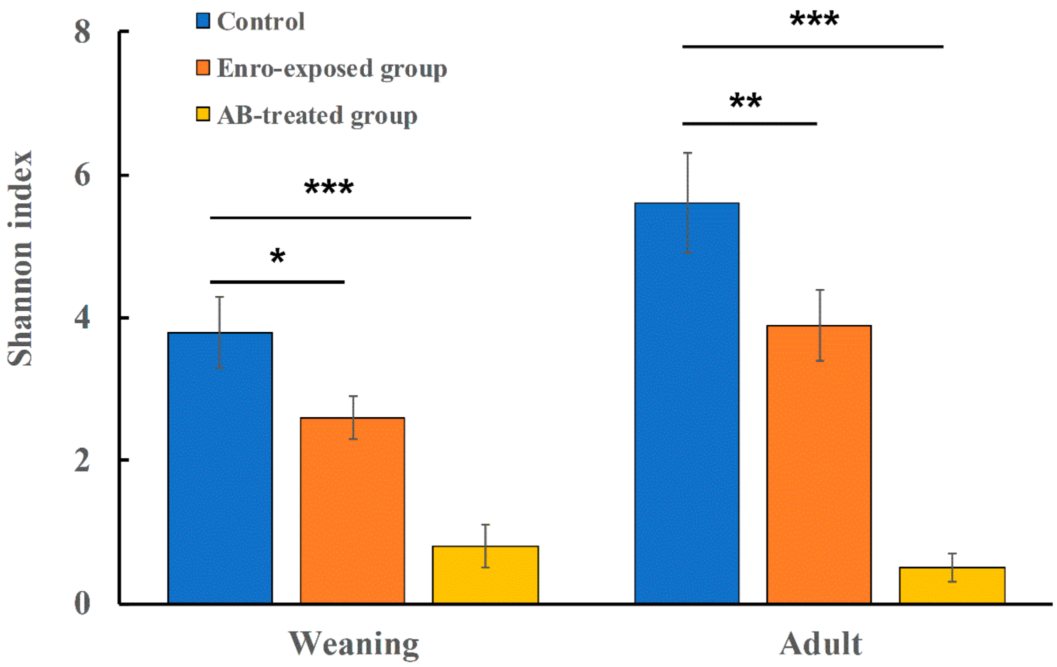

Figure S2. Alpha diversity (Shannon index) of gut microbiota in Control, Enro-exposed, and AB-treated offspring at weaning (3 weeks) and adulthood (16 weeks). Data are presented as mean ± SD, n = 5 per group. *P < 0.05, **P < 0.01, ***P < 0.001 vs. Control group at the same time point.

Supplementary Methods and Results: Verification of Gut Microbiota Depletion by Antibiotic Cocktail

Methods

Total genomic DNA was extracted from fecal samples (approximately 100 mg per sample) using the QIAamp PowerFecal Pro DNA Kit (Qiagen, Hilden, Germany) according to the manufacturer's instructions. The DNA concentration and purity were determined using a NanoDrop 2000 spectrophotometer (Thermo Fisher Scientific, Waltham, MA, USA). To assess bacterial load after antibiotic cocktail treatment, real-time quantitative PCR (qPCR) targeting the universal bacterial 16S rRNA gene was performed using the SYBR Premix Ex Taq II Kit (Takara Biomedical Technology, Beijing, China) on a LightCycler® 96 Instrument (Roche, Basel, Switzerland) with the universal bacterial primers 926F (5'-AAACTCAAAKGAATTGACGG-3') and 1062R (5'-CTCACRRCACGAGCTGAC-3'). Each 20 μL reaction contained 10 μL of SYBR Premix Ex Taq II, 0.4 μL of each forward and reverse primer (10 μM), 1 μL of template DNA (normalized to equal concentration), and 8.2 μL of sterile double-distilled water. The qPCR program consisted of an initial pre-denaturation at 95 °C for 30 s, followed by 40 cycles of denaturation at 95 °C for 5 s and annealing/extension at 60 °C for 30 s. A melting curve analysis was performed from 60 °C to 95 °C to verify the specificity of PCR products. A positive control and a no-template negative control were included in each run, and all samples were analyzed in technical triplicates. Threshold cycle (Ct) values were automatically calculated by the instrument based on the amplification

curves. Bacterial load was compared between groups based on Ct values, where a higher Ct value indicates lower 16S rRNA gene abundance and thus reduced bacterial load.

Results

To verify the efficacy of gut microbiota depletion, fecal samples were collected from the AB-treated group before antibiotic treatment (baseline, day 0) and at five time points after treatment initiation (week 1, 4, 8, 12, and 16). At baseline (day 0, prior to antibiotic treatment), high bacterial abundance was confirmed by all assays as expected: total fecal DNA concentration was 8.8 ng/mg stool (Figure S3A), and the mean Ct value of the bacterial 16S rRNA gene was 21.6 (Figure S3B). After one week of antibiotic cocktail treatment, total fecal DNA concentration decreased significantly by over 95% compared with the pre-treatment baseline (from 8.8 to 0.4 ng/mg stool, $P < 0.0001$). The mean Ct value of the bacterial 16S rRNA gene reached 38.2 at week 1, corresponding to an approximately 100,000-fold reduction in bacterial 16S rRNA gene abundance compared with baseline ($P < 0.0001$). At all five time points examined from week 1 to week 16, total fecal DNA concentration in the AB-treated group remained consistently and significantly lower than the pre-treatment baseline (0.2–0.4 ng/mg stool, corresponding to a 95–98% reduction), and Ct values remained elevated (37.5–39.0), confirming sustained microbiota depletion throughout the experimental period. These results demonstrate that the antibiotic cocktail effectively achieved gut microbial decontamination within one week of treatment.

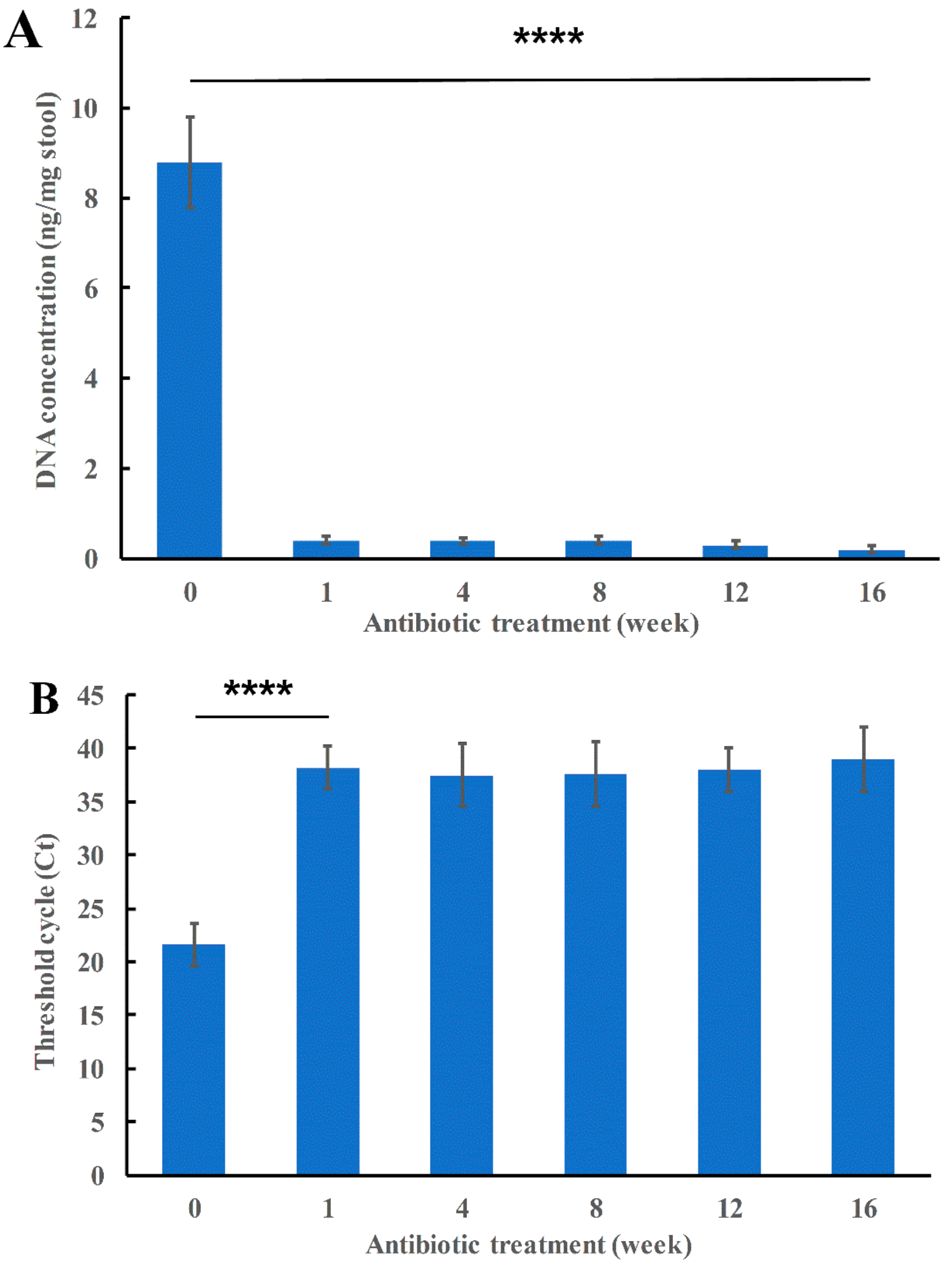

Figure S3. Verification of gut microbiota depletion by antibiotic cocktail treatment.

(A) Longitudinal changes in total fecal DNA concentration in the AB-treated group across six time points: pre-treatment baseline (week 0) and five time points after antibiotic cocktail treatment initiation (week 1, 4, 8, 12, and 16), showing sustained reduction in total fecal DNA throughout the experimental period. Data are presented as mean ± SD, n = 5 per time point. **(B)** Longitudinal changes in threshold cycle (Ct) values of the bacterial 16S rRNA gene in fecal samples from the AB-treated group across the same six time points (week 0, 1, 4, 8, 12, and 16), showing sustained elevation in Ct values (indicating reduced bacterial abundance) throughout the experimental period. Data are presented as mean ± SD, n = 5 per time point. Higher Ct values indicate lower bacterial 16S rRNA gene abundance.
